# Supplementary material for: Rational design of highly potent broad-spectrum enterovirus inhibitors targeting the nonstructural protein 2C
Source: PLoS Biol. 2020 Nov 6;18(11):e3000904. doi: 10.1371/journal.pbio.3000904 (PMC7673538; doi:10.1371/journal.pbio.3000904)
Supplement: S1 Text — (DOCX) [file pbio.3000904.s001.docx]

**Synthesis of compound 1 analogues**

Different structural modifications of compound **1** were planned in order to investigate whether its antiviral activity could be enhanced and its antiviral spectrum could be broadened. An initial effort was focused on replacing the original furan with different heteroaromatic rings and diverse heterocyclic/aliphatic groups (Fig 1B). The role of the amide bond was also explored by replacing it with either a sulfonamide bond or a methylene bridge. Different substituents on the 4-position on both rings of the *N*-benzylaniline moiety were also investigated (Fig 1C). Preparation of compound **1** analogues, in which the furan ring and the amide bond were modified, was performed through an efficient two-step synthetic pathway in which compound **4,** prepared by reductive amination between 4-fluotobenzaldehyde **2** and p-anisidine **3**, was used as common synthetic intermediate. Derivatives **5a-d** were synthesized reacting **4** with different acyl or sulfonyl chloride in dichloromethane using trimethylamine as base. Compound **1** and derivatives **5e** and **5f**, presenting a pyridine and a tetrahydrofuran ring in place of furan respectively, were obtained through an amide coupling reaction between **4** and the corresponding carboxylic acid in dimethylformamide, using DIPEA as base and TBTU as coupling agent, Reductive amination between **4** and furan-2-carbaldehyde yield compound **5g,** whereas preparation of compound **6** was achieved by two steps: amide bond formation reacting **4** and 2-bromoacetyl chloride, followed by nucleophilic displacement of the bromine atom by methyl amine. Different attempts were made for the preparation of compounds **12a-b,** in which the *N*-(4-fluoro) benzylaniline portion is bound to position three of a pyrrole ring. Coupling reaction either using TBTU or CDI as coupling agent did not give the desired product, with formation of several undesired species. After failing in converting the pyrrole-3-carboxylic acid to the corresponding acyl chloride using thionyl chloride in dichloromethane, a different approach was applied as reported in S1_Fig route **b**. The pyrrole-3-carboxylic acid nitrogen was selectively Boc-protected through a 3-step synthesis, and the resulting compounds **10a-b** were then converted into **11a-b** via TBTU-assisted coupling reaction. Removal of the Boc protecting group using TFA in dichloromethane gave compounds **12a-b** in a very high yield. Reductive amination between 4-methoxyaniline **3** and furan-2-carbaldehyde **13**, followed by the reaction with 4-fluorobenzoyl chloride gave the final product **15** in a quantitative yield. Analogues **19a-i**, bearing different substituents in 4-position of the *N*-benzylaniline moiety, were prepared following the same synthetic pathway adopted for derivatives **5a-d**. Reductive amination between differently substituted benzaldehydes and anilines yielded the intermediates **18a-I** in a high yield, which were then converted into the corresponding final compounds **19a-i** by reaction with furan-2-carbonyl chloride in dichloromethane and trimethylamine as base. 1*H*-pyrrole-2-carbonyl chloride **21**, required for the preparation of compound **22**, was prepared *in situ* by refluxing 1*H*-pyrrole-2-carboxylic acid **20** with thionyl chloride, as reported in S2 Fig.

**General chemistry methods**

All solvents and reagents used were obtained from commercial sources unless otherwise indicated. All reactions were performed under a nitrogen atmosphere. ^1^H, ^13^C and ^19^F NMR spectra were recorded with a Bruker Avance III HD spectrometer operating at 500 MHz for ^1^H, 125 MHz for ^13^C and 470 MHz for ^19^F with Me_4_Si as internal standard. Deuterated dimethyl sulfoxide (DMSO) and deutrated chloroform (CDCl_3_) were used as solvents for NMR experiments, unless otherwise stated. ^1^H chemical shifts values (δ) are referenced to the residual non-deuterated components of the NMR solvents (δ = 2.50 ppm for DMSO and 7.26 for CHCl_3_). The ^13^C chemical shifts (δ) are referenced to DMSO or CHCl_3_. Thin layer chromatography (TLC) was performed on Silica gel plates (Merck Kieselgel 60 F254), which were developed by the ascending method. Column chromatography was performed on an Isolera Biotage system. UPLC-UV-MS analysis was conducted on a Waters UPLC system with both Diode Array detection and Electrospray (+’ve and –‘ve ion) MS detection. The following conditions were applied: Waters Acquity UPLC BEH C18 1.7 µm 2.1x50 mm column, 0.5 mL/min, column temperature 40°C; mobile phase was LC-MS grade H_2_O containing 0.1% formic acid (A) and LC-MS grade MeCN containing 0.1% formic acid (B); sample diluent: MeCN; sample concentration: 1µg/mL; injection volume: 2 µL, gradient 90% eluent A (0.1 min), 90%-0% eluent A (1.5 min), 0% eluent A (1.4 min), 90% eluent A (0.1 min) (method 1). All compounds tested in biological assays were >95% pure. Purity of intermediates was >90%, unless otherwise stated.

**General procedure A:** Reductive amination for the preparation of compounds **4, 14, 18a-i**

A solution of the corresponding aniline (1.1 equiv) in a mix of anhydrous methanol (24 mL) and anhydrous tetrahydrofuran (6 mL) was treated with the corresponding benzaldehyde (1 equiv) under nitrogen atmosphere. The reaction was stirred at room temperature for 6 hours. The mixture was then cooled to 0˚ C and sodium borohydride (2 equiv) was added in small portions. The reaction was stirred at room temperature overnight. The reaction was concentrated under reduced pressure and the crude product was purified by automated flash column chromatography (*n*-hexane:EtOAc 100:0 v/v increasing to 60:40 v/v).

***N*-(4-Fluorobenzyl)-4-methoxyaniline (4)**

Reagents: *p*-anisidine and 4-fluotobenzaldehyde. Yield quantitative, brown solid. ^1^H-NMR (CDCl_3_): δ 7.37-7.32 (m, 2H, H-aromatic), 7.03 (apparent t, J_1_=8.7 Hz, 2H, H-aromatic), 6.79 (d, J=9.0 Hz, 2H, H-aromatic), 6.60 (d, J=9.0 Hz, 2H, H-aromatic), 4.26 (s, 2H, CH_2_), 3.78 (bs, 1H, NH), 3.75 (s, 3H, CH_3_). This compound was previously reported and the spectral data agree with those specified in literature.^3^

***N*-(Furan-2-ylmethyl)-4-methoxyaniline (14)**

Reagents: *p*-anisidine and furfural. Yield 88%, orange waxy solid. ^1^H-NMR (CDCl_3_): δ 7.36 (dd, J_1_=1.8 Hz, J_2_=0.8 Hz, 1H, H-aromatic), 6.79 (d, J=9.0 Hz, 2H, H-aromatic), 6.65 (d, J=9.0 Hz, 2H, H-aromatic), 6.31 (dd, J_1_=3.1 Hz, J_2_=1.8 Hz, 1H, H-aromatic), 6.21 (dd, J_1_=3.1 Hz, J_2_=0.8 Hz, 1H, H-aromatic), 4.27 (s, 2H, CH_2_), 3.76 (bs,1H, NH), 3.74 (s, 3H, CH_3_). This compound was previously reported and the spectral data agree with those specified in literature.^4^

***N*-Benzylaniline (18a)**

Reagents: aniline and benzaldehyde. Yield 96%, white waxy solid. ^1^H-NMR (CDCl_3_): δ 7.40-7.34 (m, 4H, H-aromatic), 7.29 (tt, J_1_=7.1 Hz, J_2_=1.6 Hz, 1H, H-aromatic), 7.21-7.17 (m, 2H, H-aromatic), 6.74 (tt, J_1_=7.1 Hz, J_2_= 1.1 Hz, 1H, H-aromatic), 6.67-6.64 (m, 2H, H-aromatic), 4.35 (s, 2H, CH_2_), 4.03 (bs, 1H, NH). This compound was previously reported and the spectral data agree with those specified in literature.^5^

***N*-(4-Fluorobenzyl)aniline (18b)**

Reagents: aniline and 4-fluorobenzaldehyde. Yield 40%, dark orange oil. ^1^H-NMR (CDCl_3_): δ 7.36-7.32 (m, 2H, H-aromatic), 7.18 (dd, J_1_=8.6 Hz, J_2_=7.3 Hz, 2H, H-aromatic), 7.06-7.00 (m, 2H, H-aromatic), 6,74 (tt, J_1_=7.7 Hz, J_2_= 7.3 Hz, 1H, H-aromatic), 6.65-6.62 (m, 2H, H-aromatic), 4.31 (s, 2H, CH_2_), 4.01 (bs, 1H, NH). This compound was previously reported and the spectral data agree with those specified in literature.^5^

***N*-Benzyl-4-methoxyaniline (18c)**

Reagents: *p*-anisidine and benzaldehyde. Yield 99%, yellow solid. ^1^H-NMR (CDCl_3_): δ 7.41-7.31 (m, 4H, H-aromatic), 7.27 (tt, J_1_=7.9 Hz, J_2_=7.1 Hz, 1H, H-aromatic), 6.82-6.74 (m, 2H, H-aromatic), 6.65-6.58 (m, 2H, H-aromatic), 4.29 (s, 2H, CH_2_), 3.78 (bs, 1H, NH), 3.74 (s, 3H, CH_3_). This compound was previously reported and the spectral data agree with those specified in literature.^5^

***N*-(4-(trifluoromethyl)benzyl)aniline (18d)**

Reagents: aniline and 4-trifluoromethylbenzaldehyde. Yield 94%, pale-yellow oil. ^1^H-NMR (CDCl_3_): δ δ 7.59 (d, J=8.2 Hz, 2H, H-aromatic), δ 7.49 (d, J=8.2 Hz, 2H, H-aromatic), δ 7.20-7.16 (m, 2H, H-aromatic), δ 6.74 (tt, J_1_=7.3 Hz, J_2_=1.06 Hz, 1H, H-aromatic), δ 4.42 (s, 2H, CH_2_), δ 4.15 (s, 1H, NH). This compound was previously reported and the spectral data agree with those specified in literature.^6^

**4-Methoxy-*N*-(4-(trifluoromethyl)benzyl)aniline (18e)**

Reagents: *p*-anisidine and 4-trifluoromethylbenzaldehyde. Yield 92%, pale-yellow oil. 1H-NMR (CDCl_3_): δ 7.58 (d, J=8.0 Hz, 2H, H-Aromatic), δ 7.47 (d, J=8.0 Hz, 2H, H-Aromatic), δ 6.77 (d, J=8.9 Hz, 2H, H-Aromatic), δ 6.57 (d, J=8.9 Hz, 2H, H-Aromatic), δ 4.36 (s, 2H, CH_2_), δ 3.88 (bs, 1H, NH), δ 3.74 (s, 3H, CH_3_). This compound was previously reported and the spectral data agree with those specified in literature.^7^

***N*-(4-(Pentafluorosulfaneyl)benzyl)aniline (18f)**

Reagents: aniline and 4-pentafluorosulfanylbenzaldehyde. Yield 74%, pale-yellow oil. ^1^H-NMR (CDCl_3_): δ 7.72 (d, J=8.7 Hz, 2H, H-Aromatic), δ 7.46 (d, J=8.7 Hz, 2H, H-Aromatic), 7.21-7.16 (m, 2H, H-Aromatic), 6.77-6.74 (m, 1H, H-Aromatic), δ 6.60 (d, J=7.6 Hz, 2H, H-Aromatic), δ 4.42 (s, 2H, CH_2_), δ 4.14 (bs, 1H, NH). ^13^C-NMR (CDCl_3_): δ 152.7-152.6 (m, C), 147.5 (C), 143.7 (C), 129.3 (CH), 127.2 (CH), 126.3-126.2 (m, CH), 118.1 (CH), 112.9 (CH), 47.4 (CH_2_). ^19^F-NMR (CDCl_3_): δ 63.1(d, J=150.0 Hz, 4F), 84.8 (q, J=147.4 Hz, 1F).

***N*-(4-Methylbenzyl)aniline (18g)**

Reagents: aniline and *p*-tolualdehyde. Yield 94%, white solid. ^1^H-NMR (CDCl_3_): δ 7.28 (d, J=7.9 Hz, 2H, H-aromatic), 7.21-7.15 (m, 4H, H-aromatic), 6.73 (tt, J_1_=7.6 Hz, J_2_=7.3 Hz, 1H, H-aromatic), 6.67- 6.28 (m, 2H, H-aromatic), 4.29 (s, 2H, CH_2_), 3.98 (bs, 1H, NH), 2.36 (s, 3H, CH_3_). This compound was previously reported and the spectral data agree with those specified in literature.^5^

***N*-(4-Isopropylbenzyl)aniline (18h)**

Reagents: aniline and 4-isopropylbenzaldehyde. Yield 97%, yellow oil. ^1^H-NMR (CDCl_3_): 7.34-7.28 (m, 2H, H-aromatic), 7.24-7.14 (m, 4H, H-aromatic), 6.75-6.69 (m, 1H, H-aromatic), 6.65 (d, J=8.2 Hz, 2H, H-aromatic), 4.29 (s, 2H, CH_2_), 3.98 (bs, 1H, NH), 2.97-2.85 (m, 1H, CH), 1.26 (dd, J_1_=6.9 Hz, J_2_=0.7 Hz, 6H, 2 · CH_3_). This compound was previously reported and the spectral data agree with those specified in literature.^8^

***N*-benzyl-4-(trifluoromethyl)aniline (18i)**

Reagents: benzaldehyde and 4-trifluoromethylaniline. Yield 43%, white solid. ^1^H-NMR (DMSO-d_6_): δ 7.39 – 7.29 (m, 6H, H-aromatic), 7.27 – 7.22 (m, 1H, H-aromatic), 6.99 (t, J = 6.0 Hz, 1H, H-aromatic), 6.67 (d, J = 8.6 Hz, 2H, H-aromatic), 4.33 (d, J = 6.0 Hz, 2H, CH_2_). ^13^C NMR (DMSO-d6): δ 152.07 (C), 139.85 (C), 128.86 (CH), 127.59 (CH), 127.56 (C), 127.28 (CH), 126.65 (q, J_C-F_ = 3.8 Hz, C), 112.06 (CH), 46.33 (CH_2_). ^19^F NMR (DMSO-d6): δ -58.88 (s, 3F). ^9^

General procedure B: Preparation of compounds **5a-d, 15, 19a-19i, 22**

The corresponding compound prepared using general procedure A (1 equiv) was dissolved using dry DCM (12 mL). Thriethylamine (2 equiv) was added to the mixture and the reaction cooled to 0°C. The corresponding acyl or sulphonyl chloride (1.6 equiv) was added drop-wise and the reaction mixture was allowed to warm to room temperature and then left stirring for 3 hours. After completion, the reaction mixture was concentrated under reduced pressure and purified by automated flash column chromatography.

***N*-(4-fluorobenzyl)-*N*-(4-methoxyphenyl)acetamide (5a)**

Purification by automated flash column chromatography (*n*-hexane:EtOAc 100:0 v/v increasing to 60:40 v/v). Yield: 83%, colourless oil. ^1^H-NMR (DMSO-d_6_): δ 7.20 (dd, J_1_ = 8.5 Hz, J_2_ = 5.7 Hz, 2H, H-aromatic), 7.10 (t, J = 8.9 Hz, 2H, H-aromatic), 7.06 (d, J = 8.9 Hz, 2H, H-aromatic), 6.91 (d, J = 8.9 Hz, 2H, H-aromatic), 4.78 (s, 2H), 3.73 (s, 3H), 1.79 (s, 3H). ^13^C-NMR (DMSO-d_6_): δ 170.0 (C), 161.7 (d, J_C-F_ =242.6 Hz, C), 158.7 (C), 135.6 (C), 134.4 (d, J_C-F_ =3.0 Hz, C), 130.5 (d, J_C-F_ =8.1 Hz, CH), 129.6 (CH), 115.4 (CH), 115.0(d, J_C-F_ =21.2 Hz), 55.7 (CH_3_), 51.5 (CH_2_), 22.7 (CH_3_). ^19^F-NMR (DMSO-d_6_): δ -115.7 (s, 1F). **UPLC-MS**: Rt: 1.81 min, MS (ESI)^+^: 274.1[M+1]^+^.

***N*-(4-fluorobenzyl)-*N*-(4-methoxyphenyl)furan-2-sulfonamide (5b)**

Purification by automated flash column chromatography (*n*-hexane:EtOAc 100:0 v/v increasing to 70:30 v/v). Yield: 99%, white solid. ^1^H-NMR (DMSO-d_6_): δ 8.09 (dd, J_1_ = 1.8 Hz, J_2_ = 0.9 Hz, 1H, H-aromatic), 7.29 – 7.24 (m, 2H, H-aromatic), 7.15 – 7.08 (m, 3H, H-aromatic), 6.97 – 6.93 (m, 2H, H-aromatic), 6.85 – 6.81 (m, 2H, H-aromatic), 6.75 (dd, J_1_ = 3.5 Hz, J_2_ = 1.8 Hz, 1H, H-aromatic), 4.81 (s, 2H, CH_2_), 3.70 (s, 3H, CH_3_). ^13^C-NMR (DMSO-d_6_): δ 162.2 (d, J_C-F_ =243.5 Hz, C), 159.1 (C), 148.2 (CH), 147.4 (C), 132.8 (d, J_C-F_ =3.0 Hz, C), 130.8 (C), 130.7 (d, J_C-F_ =8.3 Hz, CH), 130.5 (CH), 118.1 (CH), 115.7 (d, J_C-F_ =21.4 Hz), 114.7 (CH), 112.2 (CH), 55.7 (CH_3_), 54.2 (CH_2_). ^19^F-NMR (DMSO-d_6_): δ -114.88 (s, 1F). **UPLC-MS**: Rt: 1.98 min, MS (ESI)^+^: 362.3[M+1]^+^.

***N*-(4-fluorobenzyl)-*N*-(4-methoxyphenyl)-1H-pyrrole-2-carboxamide (5c)**

Purification by automated flash column chromatography (*n*-hexane:EtOAc 100:0 v/v increasing to 70:30 v/v). Yield: 63%, white solid. ^1^H-NMR (DMSO-d_6_): δ 11.53 (s, 1H, H-aromatic), 7.28 (dd, J_1_ = 8.7 Hz, J_2_ = 5.6 Hz, 2H, H-aromatic), 7.12 (t, J = 8.7 Hz, 2H, H-aromatic), 7.02 (d, J = 9.0 Hz, 2H, H-aromatic), 6.93 (d, J = 9.0 Hz, 2H, H-aromatic), 6.80 (td, J_1_ = 2.8 Hz, J_2_ = 1.4 Hz, 1H, H-aromatic), 5.81 (dt, J_1_ = 3.8 Hz, J_2_ = 2.5 Hz, 1H, H-aromatic), 4.92 (s, 2H, CH_2_), 4.80 (td, J_1_ = 3.7 Hz, J_2_ = 1.4 Hz, 1H, H-aromatic), 3.76 (s, 3H, CH_3_). ^13^C-NMR (DMSO-d_6_): δ 161.7 (d, J_C-F_ = 242.6 Hz, C) (C), 161.4 (C), 159.1 (C), 135.6 (C), 134.6 (d, J_C-F_ =2.9 Hz, C), 130.7 (d, J_C-F_ =8.2 Hz, CH), 130.4 (CH), 124.9 (C), 122.0 (CH), 115.5 (d, J_C-F_ =21.3 Hz, CH), 115.0 (CH), 113.7 (CH), 109.2 (CH), 55.7 (CH_3_), 52.91 (CH_2_). ^19^F-NMR (DMSO-d_6_): δ -115.63 (s, 1F). **UPLC-MS**: Rt: 1.92 min, MS (ESI)^+^: 325.2[M+1]^+^.

***N*-(4-Fluorobenzyl)-*N*-(4-methoxyphenyl)thiophene-2-carboxamide (5d)**

Purification by automated flash column chromatography (*n*-hexane:EtOAc 100:0 v/v increasing to 40:60 v/v). Yield 79%, solid. ^1^H-NMR (CDCl_3_): δ 7.30 (dd, J_1_=4.9 Hz, J_2_=1.2 Hz, 1H, H-aromatic), 7.26-7.22 (m, 2H, H-aromatic), 6.97-6.91 (m, 4H, H-aromatic), 685-6.82 (m, 3H, H-aromatic), 6.81-6.79 (m, 1H, H-aromatic), 4.96 (s, 2H, CH_2_), 3.81 (s, 3H, CH_3_). ^13^C-NMR (CDCl_3_): δ 163.2-162.2 (d, J_C-F_= 246.5 Hz, C), 162.4 (C=O), 159.5 (C), 137.9 (C), 134.8 (C), 133.1 (C), 132.7 (CH), 131.1 (CH), 130.9-130.8 (d, J_C-F_= 8.1 Hz, CH), 130.3 (CH), 126.7 (CH), 115.3-115.1 (d, J_C-F_= 21.3 Hz, CH), 114.7 (CH), 55.4 (CH_3_), 54.0 (CH_2_). ^19^F-NMR (CDCl_3_): δ -114.9 (s, 1F). **UPLC-MS**: Rt: 1.99 min, MS (ESI)^+^: 342.2 [M+H]^+^.

**4-Fluoro-*N*-(furan-2-ylmethyl)-*N*-(4-methoxyphenyl)benzamide (15)**

Purification by automated flash column chromatography (*n*-hexane:DCM 75:25 v/v increasing to 0:100 v/v). Yield quantitative, orange oil. ^1^H-NMR (CDCl_3_): δ 7.34 (dd, J_1_=1.8 Hz, J_2_=0.8 Hz, 1H, H-aromatic), 7.34-7.28 (m, 2H, H-aromatic), 6.89-6.81 (m, 4H, H-aromatic), 6.69 (d, J=8.9 Hz, 2H, H-aromatic), 6.28 (dd, J_1_=3.2 Hz, J_2_=1.8 Hz, 1H, H-aromatic), 6.22 (collapsed, 1H, H-aromatic), 5.01 (s, 2H, CH_2_), 3.73 (s, 3H, CH_3_). ^13^C-NMR (CDCl_3_): δ 169.3 (C=O), 163.1(d, J_C-F_= 250.2 Hz, C), 158.2 (C), 150.7 (C), 142.1 (CH), 136.0 (m, C), 131.8 (d, J_C-F_ =3.3 Hz, C), 131.0 (d, J_C-F_ =8.5 Hz, CH), 128.9 (CH), 114.7 (d, J_C-F_ =21.7 Hz), 114.3 (CH), 110.4 (CH), 109.1 (CH), 55.3 (CH_3_), 46.9 (CH_2_). ^19^F-NMR (CDCl_3_): δ -114.9 (s, 1F). **UPLC-MS**: Rt: 1.91 min, MS (ESI)^+^: 326.2 [M+H]^+^.

***N*-Benzyl-*N*-phenylfuran-2-carboxamide (19a)**

Purification by automated flash column chromatography (*n*-hexane:EtOAc 100:0 v/v increasing to 0:100 v/v). Yield 84%, white solid. ^1^H-NMR (CDCl_3_): δ 7.33-7.29 (m, 4H, H-aromatic), 7.29-7.26 (m, 4H, H-aromatic), 7.25-7.21 (m, 1H, H-aromatic), 7.04-6.99 (m, 2H, H-aromatic), 6.17 (dd, J_1_=3.5 Hz, J_2_=1.7 Hz, 1H, H-aromatic), 5.72 (d, J=3.5 Hz, 1H, H-aromatic), 5.04 (s, 2H, CH_2_). ^13^C-NMR (CDCl_3_): δ 159.0 (C=O), 146.8 (C), 144.3 (CH), 142.3 (C), 137.0 (C), 129.3 (CH), 128.8 (CH), 128.4 (CH), 128.3 (CH), 127.9 (CH), 127.3 (CH), 116.4 (CH), 110.8 (CH), 53.9 (CH_2_). **UPLC-MS**: Rt: 2.21 min, MS (ESI)^+^: 278.1 [M+H]^+^.

***N*-(4-Fluorobenzyl)-*N*-phenylfuran-2-carboxamide (19b)**

Purification by automated flash column chromatography (*n*-hexane:EtOAc 100:0 v/v increasing to 0:100 v/v). Yield 92%, white solid. ^1^H-NMR (CDCl_3_): δ 7.35-7.30 (m, 4H, H-aromatic), 7.25-7.21 (m, 2H, H-aromatic), 7.05-6.97 (m, 2H, H-aromatic), 6.99-6.94 (m, 2H, H-aromatic), 6.17 (dd, J_1_=3.5 Hz, J_2_=1.7 Hz, 1H, H-aromatic), 5.71 (d, J=3.4 Hz, 1H, H-aromatic), 5.00 (s, 2H, CH_2_). ^13^C-NMR (CDCl_3_): δ 162.2 (d, J_C-F_= 245.7 Hz, C), 159.1 (C=O), 146.8 (C), 144.5 (CH), 142.1 (C), 132.8 (d, J_C-F_= 3.2 Hz, C), 130.7 (d, J_C-F_=8.1 Hz, CH), 129.5 (CH), 128.5 (CH), 128.2 (CH), 116.6 (CH), 115.2 (d, J_C-F_= 21.3 Hz, CH), 110.0 (CH), 53.3 (CH_2_). ^19^F-NMR (CDCl_3_): δ -114.9 (s, 1F). **UPLC-MS**: Rt: 1.90 min, MS (ESI)^+^: 296.0 [M+H]^+^.

***N*-Benzyl-*N*-(4-methoxyphenyl)furan-2-carboxamide (19c)**

Purification by automated flash column chromatography (*n*-hexane:DCM 75:25 v/v increasing to 0:100 v/v). Yield 90%, white solid. ^1^H-NMR (CDCl_3_): δ 7.36 (dd, J_1_=1.7 Hz, J_2_=0.7 Hz, 1H, H-aromatic), 7.28-7.26 (m, 4H, H-aromatic), 7.25-7.21 (m, 1H, H-aromatic), 6.91 (d, J=9.0 Hz, 2H, H-aromatic), 6.81 (d, J=9.0 Hz, 2H, H-aromatic), 6.18 (dd, J_1_=3.5 Hz, J_2_=1.7 Hz, 1H, H-aromatic), 5.63 (bs, 1H, H-aromatic), 4.99 (s, 2H, CH_2_), 3.80 (s, 3H, CH_3_). ^13^C-NMR (CDCl_3_): δ 159.3 (C=O), 159.2 (C), 146.9 (C), 144.4 (CH), 137.2 (C), 134.9 (C), 129.7 (CH), 129.1 (CH), 128.3 (CH), 127.4 (CH), 116.4 (CH), 114.5 (CH), 111.0 (CH), 55.4 (CH_3_), 54.1 (CH_2_). **UPLC-MS**: Rt: 2.17 min, MS (ESI)^+^: 308.1 [M+H]^+^.

***N*-phenyl-*N*-(4-(trifluoromethyl)benzyl)furan-2-carboxamide (19d)**

Purification by automated flash column chromatography (n-hexane:EtOAc 100:0 v/v increasing to 70:30 v/v). Yield 88%, yellow solid. ^1^H-NMR (DMSO-d_6_): δ 7.72 – 7.64 (m, 3H, H-aromatic), 7.50 (d, J = 8.0 Hz, 2H, H-aromatic), 7.41 – 7.30 (m, 3H, H-aromatic), 7.20 (d, J = 7.0 Hz, 2H, H-aromatic), 6.40 (dd, J_1_ = 3.5 Hz, J_2_ = 1.7 Hz, 1H, H-aromatic), 5.89 (d, J = 3.3 Hz, 1H, H-aromatic), 5.11 (s, 2H, CH_2_). ^13^C-NMR (DMSO-d_6_): δ 159.1 (C=O), 146.8 (C), 142.5 (C), 129.9 (CH), 129.1 (CH), 128.3 (CH), 128.3 (CH), 125.7 (q, J_C-F_ = 3.7 Hz, CH), 117.0 (CH), 111.8 (CH), 53.1 (CH_2_). ^19^F-NMR (DMSO-d_6_): δ -60.86 (s, 3F). **UPLC-MS**: Rt: 2.03 min, MS (ESI)^+^: 346.2[M+1]^+^.

***N*-(4-Methoxyphenyl)-*N*-(4-(trifluoromethyl)benzyl)furan-2-carboxamide (19e)**

Purification by automated flash column chromatography (*n*-hexane:EtOAc 100:0 v/v increasing to 20:80 v/v). Yield 66%, pale-yellow oil. ^1^H-NMR (CDCl_3_): δ 7.52 (d, J=8.2 Hz, 2H, H-aromatic), 7.40 (d, J=8.2 Hz, 2H, H-aromatic), 7.38-7.37 (m, 1H, H-aromatic), 6.92 (d, J=8.9 Hz, 2H, H-aromatic), 6.84 (d, J=8.9 Hz, 2H, H-aromatic), 6.20 (dd, J_1_=3.5 Hz, J_2_=1.7 Hz, 1H, H-aromatic), 5.69-5.65 (m, 1H, H-aromatic), 5.04 (s, 2H, CH_2_), 3.81 (s, 3H, CH_3_). ^13^C-NMR (CDCl_3_): δ 159.4 (C), 159.3 (C=O), 146.6 (C), 144.7 (CH), 141.3-141.2 (m, C), 134.7 (C), 129.8 (q, J_C-F_= 32.3 Hz, C), 129.5 (CH), 129.3 (CH), 125.3 (q, J_C-F_= 3.7 Hz, CH), 125.2 (q, J_C-F_= 271.9 Hz, C), 116.7 (CH), 114.7 (CH), 111.1 (CH), 55.4 (CH_3_), 53.8 (CH_2_). ^19^F-NMR (CDCl_3_): δ -62.49 (s, 3F). **UPLC-MS**: Rt: 2.02 min, MS (ESI)^+^: 376.3 [M+H]^+^.

***N*-(4-(Pentafluorosulfaneyl)benzyl)-N-phenylfuran-2-carboxamide (19f)**

Purification by automated flash column chromatography (*n*-hexane:DCM 100:0 v/v increasing to 0:100 v/v). Yield 56%, pale-yellow oil. ^1^H-NMR (CDCl_3_): δ 7.66 (d, J=8.8 Hz, 2H, H-aromatic), 7.40 (d, J=8.8 Hz, 2H, H-aromatic), 7.38-7.35 (m, 3H, H-aromatic), 7.34 (dd, J_1_=1.7 Hz, J_2_=0.9 Hz, 1H, H-aromatic), 7.08-7.03 (m, 2H, H-aromatic), 6.21 (dd, J_1_=3.5 Hz, J_2_=1.7 Hz, 1H, H-aromatic), 5.79 (d, J=3.5 Hz, 1H, H-aromatic), 5.06 (s, 2H, CH_2_). ^13^C-NMR (CDCl_3_): δ 159.3 (C=O), 153.0 (m, C), 146.5 (C), 144.7 (CH), 142.2 (C), 141.0 (C), 129.7 (CH), 129.0 (CH), 128.3 (CH), 128.2 (CH), 126.1-126.0 (m, CH), 117.0 (CH), 111.1 (CH), 53.4 (CH_3_). ^19^F-NMR (CDCl_3_): δ 62.8(d, J=150.0 Hz, 4F), 84.6 (q, J=147.4 Hz, 1F). **UPLC-MS**: Rt: 2.06 min, MS (ESI)^+^: 404.2 [M+H]^+^.

***N*-(4-Methylbenzyl)-N-phenylfuran-2-carboxamide (19g)**

Purification by automated flash column chromatography (*n*-hexane:DCM 75:25 v/v increasing to 0:100 v/v). Yield 67%, white solid. ^1^H-NMR (CDCl_3_): δ 7.35-7.29 (m, 4H, H-aromatic), 7.16 (d, J=7.9 Hz, 2H, H-aromatic), 7.06 (d, J=7.9 Hz, 2H, H-aromatic), 7.04-6.98 (m, 2H, H-aromatic), 6.17 (dd, J_1_=3.5 Hz, J_2_=1.7 Hz, 1H, H-aromatic), 5.70 (d, J=3.5 Hz, 1H, H-aromatic), 5.00 (s, 2H, CH_2_), 2.30 (s,3H, CH_3_). ^13^C-NMR (CDCl_3_): δ 159.1 (C=O), 147.0 (C), 144.4 (CH), 142.4 (C), 137.1 (C), 134.0 (C), 129.3 (CH), 129.0 (CH), 128.9 (CH), 128.5 (CH), 128.0 (CH), 116.4 (CH), 110.9 (CH), 53.7 (CH_2_), 21.1 (CH_3_). **UPLC-MS**: Rt: 1.98 min, MS (ESI)^+^: 292.0 [M+H]^+^.

***N*-(4-Isopropylbenzyl)-N-phenylfuran-2-carboxamide (19h)**

Purification by automated flash column chromatography (*n*-hexane:DCM 75:25 v/v increasing to 0:100 v/v). Yield 99%, white solid. ^1^H-NMR (CDCl_3_): δ 7.37-7.28 (m, 4H, H-aromatic), 7.19 (d, J=8.2 Hz, 2H, H-aromatic), 7.12 (d, J=8.2 Hz, 2H, H-aromatic), 7.06-7.00 (m, 2H, H-aromatic), 6.17 (dd, J_1_=3,3 Hz, J_2_=1.7 Hz, 1H, H-aromatic), 5.71 (d, J=3.3 Hz, 1H, H-aromatic), 5.00 (s, 2H, CH_2_), 2.92-2.80 (m, 1H, CH), 1.21 (d, J=6.9 Hz, 6H, 2 · CH_3_). ^13^C-NMR (CDCl_3_): δ 159.1 (C=O), 148.1 (C), 147.0 (C), 144.3 (CH), 142.6 (C), 134.4 (C), 129.3 (CH),128.9 (CH), 128.5 (CH), 127.9 (CH), 126.4 (CH), 116.4 (CH), 110.9 (CH), 53.8 (CH_2_), 33.8 (CH), 24.0 (2 ·CH_3_). **UPLC-MS**: Rt: 2.12 min, MS (ESI)^+^: 320.2 [M+H]^+^.

***N*-benzyl-*N*-(4-(trifluoromethyl)phenyl)furan-2-carboxamide (19i)**

Purification by automated flash column chromatography (*n*-hexane:DCM 100:0 v/v increasing to 0:100 v/v). Yield 99%, yellow solid. ^1^H-NMR (DMSO-d_6_): δ δ 7.70 (d, J = 8.4 Hz, 2H, H-aromatic), 7.66 (dd, J_1_ = 1.7 Hz, J_2_ = 0.8 Hz, 1H, H-aromatic), 7.38 (d, J = 8.2 Hz, 2H, H-aromatic), 7.33 – 7.20 (m, 5H, H-aromatic), 6.47 (dd, J_1_ = 3.5 Hz, J_2_ = 1.7 Hz, 1H, H-aromatic), 6.35 (dd, J_1_ = 3.5 Hz, J_2_ = 0.7 Hz, 1H, H-aromatic), 5.10 (s, 2H, CH_2_). ^13^C-NMR (DMSO-d_6_): δ 159.1 (C=O), 147.0 (C), 146.4 (C), 145.9 (CH), 137.3 (C), 128.9 (CH), 128.5 (CH), 128.3 (CH), 127.8 (q, J_C-F_ = 32.1 Hz, C), 127.8 (CH), 126.7 (q, J_C-F_ = 3.7 Hz, CH), 124.4 (q, J_C-F_ = 272.2 Hz, C), 117.5 (CH), 112.0 (CH), 53.1 (CH_2_). ^19^F-NMR (DMSO-d_6_): δ -60.81 (s, 3F). **UPLC-MS**: Rt: 2.059 min, MS (ESI)^+^: 346.2[M+1]^+^.

***N*-Phenyl-*N*-(4-(trifluoromethyl)benzyl)-1H-pyrrole-2-carboxamide (22)**

The required 1*H*-pyrrole-2-carbonyl chloride (**21**) was prepared *in situ* by refluxing 1*H*-pyrrole-2-carboxylic acid (**20**) (1 equiv) with thionyl chloride (1 equiv) in dry dichloromethane (6 mL) for two hours. After removing the solvent, the obtained chloride was used without any further purification for the preparation of compound 22. Purification by automated flash column chromatography (*n*-hexane:EtOAc 100:0 v/v increasing to 40:60 v/v). Yield 75%, pale-yellow oil. ^1^H-NMR (CDCl_3_): δ 9.61 (s, 1H, NH), 7.54 (d, J=8.0 Hz, 2H, H-aromatic), 7.43-7.37 (m, 5H, H-aromatic), 7.12-7.0 (m, 2H, H-aromatic), 6.84-6.83(m, 1H, H-aromatic), 5.92-5.90 (m, 1H, H-aromatic), 5.07 (s, 2H, CH_2_), 4.88-4.87 (m, 1H, H-aromatic). ^13^C-NMR (CDCl_3_): δ 148.3 (C=O), 142.4 (C), 141.5 (m, C), 129.7 (CH), 129.5 (C), 129.9 (CH), 128.8 (CH), 128.6 (CH), 125.4 (q, J_C-F_= 3.7 Hz, CH), 125.2 (q, J_C-F_= 271.1 Hz, C), 124.6 (C), 121.2 (CH), 114.1 (CH), 109.9 (CH), 53.8 (CH_2_). ^19^F-NMR (CDCl_3_): δ -62.48 (s, 3F). **UPLC-MS**: Rt: 2.05 min, MS (ESI)^+^: 345.2 [M+H]^+^.

General procedure C: Preparation of compounds **1, 5e-f**

The corresponding compound prepared using general procedure A (1 equiv) and TBTU (1.2 equiv) were suspended in dry DMF (5 mL) under nitrogen atmosphere. The corresponding acid (1.1 equiv) was added. The reaction mixture was cooled to 0° C in an-ice bath and then DIPEA (3.5 equiv) was added. The reaction was stirred at room temperature overnight. After completion, the reaction mixture was concentrated under vacuum and the residue dissolved with EtOAc (35 mL) and washed with HCl 1M (25 mL), NaHCO_3_ (25 mL) and brine (25 mL). The organic phase was dried over Na_2_SO_4_, concentrated under vacuum and the crude product purified by automated flash column chromatography (*n*-hexane:DCM 75:25 v/v increasing to 0:100 v/v).

***N*-(4-fluorobenzyl)-*N*-(4-methoxyphenyl)furan-2-carboxamide (1)**

Purification by automated flash column chromatography (*n*-hexane:EtOAc 100:0 v/v increasing to 70:30 v/v). Yield 60%, white solid. ^1^H-NMR (DMSO-d_6_): δ 7.71 – 7.67 (m, 1H, H-aromatic), 7.27 (dd, J_1_ = 8.7 Hz, J_2_ = 5.6 Hz, 2H, H-aromatic), 7.12 (t, J = 8.7 Hz, 2H, H-aromatic), 7.03 (d, J = 8.9 Hz, 2H, H-aromatic), 6.91 (d, J = 8.9 Hz, 2H, H-aromatic), 6.39 (dd, J_1_ = 3.5 Hz, J_2_ = 1.7 Hz, 1H, H-aromatic), 5.72 (apparent s, 1H, H-aromatic), 4.93 (s, 2H, CH_2_), 3.74 (s, 3H, CH_3_). ^13^C-NMR (DMSO-d_6_): δ 161.8 (d, J_C-F_ =242.9 Hz, C), 159.0 (C=O), 158.9 (C), 146.9 (CH), 145.6 (C), 134.9 (C), 133.9 (d, J_C-F_ =3.0 Hz, C), 130.8 (d, J_C-F_ =8.2 Hz, CH), 129.8 (CH), 116.6 (CH), 115.6 (d, J_C-F_ =21.3 Hz, CH), 115.0 (CH), 111.7 (CH), 55.7 (CH_3_), 52.8 (CH_2_). ^19^F-NMR (DMSO-d_6_): δ -115.39 (s, 1F). **UPLC-MS**: Rt: 1.92 min, MS (ESI)^+^: 326.2[M+1]^+^, 348.2[M+Na]+.

***N*-(4-fluorobenzyl)-*N*-(4-methoxyphenyl)picolinamide (5e)**

Purification by automated flash column chromatography (DCM:MeOH 100:0 v/v increasing to 99:1 v/v). Yield 37%, white solid. ^1^H-NMR (DMSO-d_6_): δ 8.32 (s, 1H, H-aromatic), 7.71 (t, J = 6.9 Hz, 1H, H-aromatic), 7.45 (d, J = 7.4 Hz, 1H, H-aromatic), 7.33 (dd, J_1_ = 8.4 Hz, J_2_ = 5.7 Hz, 2H, H-aromatic), 7.23 (s, 1H, H-aromatic), 7.15 (t, J = 8.5 Hz, 2H, H-aromatic), 6.89 (d, J = 8.0 Hz, 2H, H-aromatic), 6.67 (d, J = 8.0 Hz, 2H, H-aromatic), 5.03 (s, 2H, CH_2_), 3.61 (s, 3H, CH_3_). ^13^C-NMR (DMSO-d_6_): δ 168.8 (C=O), 161.8 (d, J_C-F_ =242.9 Hz, C), 157.9 (C), 155.0 (C), 148.7 (CH), 136.9 (CH), 135.0 (C), 133.9 (d, J_C-F_ =3.0 Hz, C), 130.5 (d, J_C-F_ =8.0 Hz, CH), 129.4 (CH), 124.2 (CH), 123.5 (CH), 115.6 (d, J_C-F_ =21.3 Hz, CH), 114.2 (CH), 55.5 (CH_3_), 52.0 (CH_2_). ^19^F-NMR (DMSO-d_6_): δ -115.44 (s, 1F). **UPLC-MS**: Rt: 1.806 min, MS (ESI)^+^: 337.3[M+1]^+^, 359.3[M+Na+]^+^

***N*-(4-Fluorobenzyl)-*N*-(4-methoxyphenyl)tetrahydrofuran-2-carboxamide (5f)**

Yield 69%, yellow oil. ^1^H-NMR (CDCl_3_): δ 7.14 (dd, J_1_=6.5 Hz, J_2_=5.4 Hz, 2H, H-aromatic), 6.95-690 (m, 2H, H-aromatic), 6.85 (d, J=9.0 Hz, 2H, H-aromatic), 6.81 (d, J=9.1Hz, 2H, H-aromatic), 4.91 (d, J=14.0 Hz, 1H, CH_2_), 4.65 (d, J=14.0 Hz, 1H, CH_2_), 4.25 (dd, J_1_=11.0 Hz, J_2_=6.0 Hz, 1H, CH_2_), 4.08-4.01 (m, 1H, CH_2_), 3.85-3.78 (m, 1H, CH), 3.78 (s,3H, CH_3_), 2.08-1.91 (m, 2H, CH_2_), 1.85-1.67 (m, 2H, CH_2_). ^13^C-NMR (CDCl_3_): δ 173.0 (C=O),162.1 (d, J_C-F_= 245.5 Hz, C), 159.1 (C), 133.6 (C), 133.1 (d, J_C-F_= 3.2 Hz, C), 130.7 (d, J_C-F_=8.1 Hz, CH), 129.7 (CH), 115.1 (d, J_C-F_= 21.3 Hz, CH), 114.6 (CH), 75.0 (CH), 69.59 (CH_2_), 55.4 (CH_3_), 52.6 (CH_2_), 30.3 (CH_2_), 25.9 (CH_2_). ^19^F-NMR (CDCl_3_): δ -115.0 (s, 1F). **UPLC-MS**: Rt: 1.84 min, MS (ESI)^+^: 330.2 [M+H]^+^.

**Preparation of *N*-(4-fluorobenzyl)-*N*-(furan-2-ylmethyl)-4-methoxyaniline (5g)**

*N*-(4-fluorobenzyl)-4-methoxyaniline (**4**) (1.8 equiv) was dissolved in methanol:tetrahydrofuran (4:1, 2 mL). Furan-2-carbaldehyde (1 equiv) was added and the mixture was stirred at room temperature for 3 hours. The reaction mixture was cooled to 0°C before adding sodium triacetoxyborohydride (2 equiv). The reaction was allowed to warm-up to room temperature and stirred overnight. After completion, the reaction mixture was concentrated under vacuum, water (30 mL) was added and the mixture was extracted with DCM (3x20 mL). The combined organic layers were washed with brine (3x20 mL), dried over Na_2_SO_4_ and concentrated under vacuum. The crude product was purified by automated flash column chromatography (*n*-hexane:EtOAc 100:0 v/v increasing to 60:40 v/v).

Yield 30%, yellow oil. ^1^H-NMR (DMSO-d_6_): δ 7.57 (dd, J_1_ = 1.8 Hz, J_2_ = 0.8 Hz, 1H, H-aromatic), 7.29 – 7.23 (m, 2H, H-aromatic), 7.11 (ddd, J_1_ = 9.6, 5.9 Hz, J_2_ = 2.6 Hz, 2H, H-aromatic), 6.74 (s, 4H, H-aromatic), 6.37 (dd, J_1_ = 3.2 Hz, J_2_ = 1.8 Hz, 1H, H-aromatic), 6.25 (dd, J_1_ = 3.2 Hz, J_2_ = 0.7 Hz, 1H, H-aromatic), 4.49 (s, 2H, CH_2_), 4.47 (s, 2H, CH_2_), 3.63 (s, 3H, CH_3_). ^13^C-NMR (DMSO-d_6_): δ 161.5 (d, J_C-F_ =242.0 Hz, C), 152.9 (C), 151.9 (C), 142.7 (C), 142.6 (CH), 135.7 (d, J_C-F_= 2.8 Hz, C), 129.2 (d, J_C-F_= 8.0 Hz, CH), 115.6 (CH), 115.5 (d, J_C-F_= 21.2 Hz, CH), 114.8 (CH), 110.7 (CH), 108.2 (CH), 55.6 (CH_3_), 54.3 (CH_2_), 48.6 (CH_2_). ^19^F-NMR (DMSO-d_6_): δ -116.47 (s, 1F). **UPLC-MS**: Rt: 2.13, MS (ESI)^+^: 312.1[M+1]^+^.

**Preparation of *N*-(4-fluorobenzyl)-*N*-(4-methoxyphenyl)-2-(methylamino)acetamide (6).**

2-bromoacetyl chloride (1 equiv) was added drop-wise to a solution of *N*-(4-fluorobenzyl)-4-methoxyaniline (**4**) (1.1 equiv) and TEA (2 equiv) in anhydrous DCM (0.2 M) at 0°C. The resulting mixture was stirred at room temperature for 3 hours. The reaction mixture was diluted in DCM and washed with a 1M HCl solution (20 mL), brine (20 mL), dried over Na2SO4and concentrated under vacuum. The crude product purified by automated flash column chromatography (DCM:MeOH 100:0 v/v increasing to 99:1 v/v).

Yield 87%, yellow oil. ^1^H-NMR (DMSO-d_6_): δ 7.25 – 7.18 (m, 2H, H-aromatic), 7.18 – 7.08 (m, 4H, H-aromatic), 6.95 – 6.84 (m, 2H, H-aromatic), 4.81 (s, 2H, CH_2_), 4.04 (s, 2H, CH_2_), 3.74 (s, 3H, CH_3_).

2-bromo-N-(4-fluorobenzyl)-N-(4-methoxyphenyl)acetamide (1 equiv) was dissolved in absolute EtOH and methylamine in EtOH (2 equiv) was added. The mixture was stirred at room temperature overnight. The solvent was evaporated under reduced pressure and the crude product was purified by automated flash column chromatography (DCM:MeOH 100:0 v/v increasing to 95:5 v/v).

Yield: 66%, yellow oil. ^1^H-NMR (DMSO-d_6_): δ 7.21 (dd, J_1_ = 8.5 Hz, J_2_= 5.7 Hz, 2H, H-aromatic), 7.11 (t, J = 8.9 Hz, 2H, H-aromatic), 7.05 (d, J = 8.9 Hz, 2H, H-aromatic), 6.92 (d, J = 8.9 Hz, 2H, H-aromatic), 4.81 (s, 2H, CH_2_), 3.74 (s, 3H, CH_3_), 3.03 (s, 2H, CH_2_), 2.22 (s, 3H, CH_3_). ^13^C-NMR (DMSO-d_6_): δ 170.7 (C=O), 161.8 (d, J_C-F_= 242.8 Hz, CH), 159.0 (C), 134.1 (d, J_C-F_= 3.0 Hz, C) 133.9 (C), 130.6 (d, J_C-F_= 8.2 Hz, CH), 129.7 (CH), 115.5 (d, J_C-F_= 21.3 Hz, CH), 115.1 (CH), 55.7 (CH_3_), 52.5 (CH_2_), 51.8 (CH_2_), 35.9 (CH_3_). ^19^F-NMR (DMSO-d_6_): δ -115.53 (s, 1F). **UPLC-MS:** Rt: 1.45, MS (ESI)^+^: 303.2[M+1]^+^.

**Preparation of Benzyl 1*H*-pyrrole-3-carboxylate (8)**

Pyrrole-3-carboxylic acid (**7**) (1 equiv) was dissolved in dimethylformamide (10 mL). K_2_CO_3_ (1.5 equiv) and benzyl bromide (1.05 equiv) were added and the mixture was stirred eighteen hours at room temperature. After completion, the reaction mixture was concentrated under vacuum, water (30 mL) was added and the mixture was extracted with EtOAc (3x40 mL). The combined organic layers were washed with water (50 mL) and brine (40 mL), dried over MgSO_4_ and concentrated under vacuum. The crude product purified by automated flash column chromatography (*n*-hexane:EtOAc 100:0 v/v increasing to 60:40 v/v).

Yield 68%, yellow oil. ^1^H-NMR (CDCl_3_): δ 8.61 (bs, 1H, NH), 7.48-7.43 (m, 1H, H-aromatic), 7.43-7.40 (m, 2H, H-aromatic), 7.39-7.34 (m, 2H, H-aromatic), 7.33-7.29 (m, 1H, H-aromatic),

6.77-6.73 (m, 1H, H-aromatic), 6.71-6.67 (m, 1H, H-aromatic), 5.29 (s, 2H, CH_2_). ^13^C-NMR (CDCl_3_): δ 164.8 (C=O), 136.7 (C), 128.5 (CH), 128.0 (CH), 127.9 (CH), 123.7 (CH), 118.8

(CH), 116.3 (C), 109.9 (CH), 65.5 (CH_2_).

**Preparation of 3-Benzyl 1-(*tert*-butyl) 1*H*-pyrrole-1,3-dicarboxylate (9)**

Benzyl 1*H*-pyrrole-3-carboxylate (**8**) (1 equiv), di-tert-butyl dicarbonate (1.15 equiv), 4-

Dimethylaminopyridine (0.09 equiv) and triethylamine (1.1 equiv) were dissolved in tetrahydrofuran (10 mL). The mixture was stirred at room temperature for eighteen hours. After completion, the reaction was concentrated under vacuum and the residue was dissolved with EtOAc (70 mL) and the organic layer was washed with HCl 0.1 N (40 mL), water (40 mL) and

brine (40 mL). The organic layer was dried over Na_2_SO_4_, concentrated under vacuum and the crude product purified by automated flash column chromatography (*n*-hexane:EtOAc 100:0 v/v increasing to 60:40 v/v).

Yield 90%, transparent oil. ^1^H-NMR (CDCl_3_): δ 7.85 (dd, J_1_=2.1 Hz, J_2_=1.6 Hz, 1H, H-aromatic), 7.44-7.39 (m, 2H, H-aromatic), 7.39-7.35 (m, 2H, H-aromatic), 7.35-7.30 (m, 1H, H-aromatic), 7.20 (dd, J_1_=3.3 Hz, J_2_=2.1 Hz, 1H, H-aromatic), 6.63 (dd, J_1_=3.3Hz, J_2_=1.6 Hz, 1H, H-aromatic), 5.29 (s, 2H, CH_2_), 1.60 (s, 9H, 3 · CH_3_). ^13^C-NMR (CDCl_3_): δ 163.9 (C=O), 148.1 (C=O), 136.3 (C), 128.5 (CH), 128.1 (CH), 125.0 (CH), 120.7 (CH), 119.2 (C), 112.0 (CH), 85.0 (C), 65.9 (CH_2_), 27.9 (3 · CH_3_).

**Preparation of 1-(*tert*-Butoxycarbonyl)-1*H*-pyrrole-3-carboxylic acid (10)**

3-Benzyl 1*-(tert*-butyl) 1*H*-pyrrole-1,3-dicarboxylate (**9**) was dissolved in mixture of ethylacetate:methanol (1:1, 11 mL). Pd/C 10% was added as catalyst and the reaction was stirred for twenty-four hours under H_2_ atmosphere. The catalyst was removed by filtration through celite pad, using a mixture of ethylacetate:methanol (1:1) to wash the pad. The filtrate was concentrated under reduced pressure and the crude product purified by automated flash column chromatography (*n*-hexane:EtOAc 100:0 v/v increasing to 0:100 v/v).

Yield 93%, yellow oil. ^1^H-NMR (CDCl_3_): δ 10.03 (bs, 1H, COOH), 7.90-7.89 (m, 1H, H-aromatic), 7.21 (dd, J_1_=3.2 Hz, J_2_=2.1 Hz, 1H, H-aromatic), 4.62 (dd, J_1_=4.9 Hz, J_2_=1.6 Hz, 1H, H-aromatic), 1.61 (s, 9H, 3 · CH_3_). ^13^C-NMR (CDCl_3_): δ 166.1 (C=O), 148.1 (C=O), 118.8 (CH), 116.1 (CH), 109.8 (C), 103.1 (CH), 84.5 (C), 27.9 (3 · CH_3_).

**Preparation of *tert*-Butyl-3-((4-fluorobenzyl)(4-methoxyphenyl)carbamoyl)-1*H*-pyrrole-1-carboxylate (11a)**

Prepared according to general procedure C. The reaction was left stirring at 45˚C for 48 hours. HCl 0.5M (25 mL) was used for the work-up. Purification by automated flash column chromatography (*n*-hexane:EtOAc 100:0 v/v increasing to 50:50 v/v). Yield 79%, orange oil. ^1^H-NMR (CDCl_3_): δ 7.25-7.21 (m, 2H, H-aromatic), 6.97-6.87 (m, 6H, H-aromatic), 6.82 (d, J=9.0 Hz, 2H, H-aromatic), 5.83 (dd, J_1_=3.3 Hz, J_2_=1.6 Hz, 1H, H-aromatic), 4.95 (s, 2H, CH_2_), 3.79 (s, 3H, CH_3_), 1.50 (s, 9H, 3 · CH_3_). ^13^C-NMR (CDCl_3_): δ 164.3 (C=O), 162.1 (d, J_C-F_= 245.3 Hz, C), 159.1 (C), 148.1 (C), 135.4 (C), 133.4 (d, J_C-F_= 3.2 Hz, C), 130.6 (d, J_C-F_=8.0 Hz, CH), 129.9 (CH), 124.3 (CH), 122.2 (C), 119.1 (CH), 115.1 (d, J_C-F_= 21.3 Hz, CH), 114.5 (CH), 113.1 (CH), 84.3 (C), 55.4 (CH_3_), 53.3 (CH_2_), 27.8 (3 · CH3). ^19^F-NMR (CDCl_3_): δ -115.0 (s, 1F). **UPLC-MS**: Rt: 2.53 min, MS (ESI)^+^: 425.3[M+H]^+^.

**Preparation of *tert*-Butyl-3-((4-fluorobenzyl)(phenyl)carbamoyl)-1*H*-pyrrole-1-carboxylate (11b)**

Prepared according to general procedure C. The reaction was left stirring at 45˚C for 72 hours. HCl 0.5M (25 mL) was used for the work-up. Purification by automated flash column chromatography (*n*-hexane:diethylether 100:0 v/v increasing to 60:40 v/v). Yield 45%, orange oil. ^1^H-NMR (CDCl_3_): δ 7.33-7.30 (m, 3H, H-aromatic), 7.25-7.22 (m, 2H, H-aromatic), 7.03-7.01 (m, 2H, H-aromatic), 6.97-6.92 (m, 3H, H-aromatic), 6.88-6.87 (m, 1H, H-aromatic), 5.82 (dd, J_1_=3.3 Hz, J_2_=1.6 Hz, 1H, H-aromatic), 5.00 (s, 2H, CH_2_), 1.50 (s, 9H, 3 · CH_3_). ^13^C-NMR (CDCl_3_): δ 165.9 (C=O), 163.1 (d, J_C-F_= 246.3 Hz, C), 148.0 (C), 142.8 (C), 134.2 (C), 133.3 (d, J_C-F_= 3.2 Hz, C), 130.5 (d, J_C-F_=8.0 Hz, CH), 129.4 (CH), 128.7 (CH), 127.9 (CH), 124.3 (CH), 122.1 (C), 119.0 (CH), 115.1 (d, J_C-F_= 21.3 Hz, CH), 113.0 (CH), 84.4 (C), 53.2 (CH_2_), 27.8 (3 · CH3). ^19^F-NMR (CDCl_3_): δ -115.2 (s, 1F). **UPLC-MS**: Rt: 2.58 min, MS (ESI)^+^: 395.0[M+H]^+^.

General procedure D: Preparation of compounds **12a-b**

Compounds **11a-b** were dissolved in dichloromethane (2.5 mL) and the mixture was cooled to 0°C in an ice-bath. A solution of dichloromethane:trifluoroacetic acid (TFA) (1:1, 2 mL) was added drop-wise and stirring at room temperature for four hours. After completion, the reaction mixture was concentrated under vacuum and the excess of TFA removed by co-evaporation with dichloromethane. The crude compound was purified by cation exchange column chromatography eluting with MeOH and then ammonia (7M in methanol).

***N*-(4-Fluorobenzyl)-*N*-(4-methoxyphenyl)-1*H*-pyrrole-3-carboxamide (12a)**

Yield 91%, brown solid. ^1^H-NMR (CDCl_3_): δ 8.27 (bs, 1H, NH), 7.26-7.21 (m, 2H, H-aromatic), 6.97-6.90 (m, 4H, H-aromatic), 6.82 (d, J=8.9 Hz, 2H, H-aromatic), 6.58-6.55 (m, 1H, H-aromatic), 6.49 (m, 1H, H-aromatic), 5.86-5.83 (m, 1H, H-aromatic), 4.97 (s, 2H, CH_2_), 3.80 (s, 3H, CH_3_). ^13^C-NMR (CDCl_3_): δ 165.3 (C=O), 162.0 (d, J_C-F_= 242.3 Hz, C), 158.9 (C), 136.1 (C), 133.8 (d, J_C-F_= 3.2 Hz, C), 130.6 (d, J_C-F_=8.1 Hz, CH), 130.0 (CH), 123.1 (CH), 119.2 (C), 117.3 (CH), 115.1 (d, J_C-F_= 21.2 Hz, CH), 114.4 (CH), 110.8 (CH), 55.4 (CH_3_), 53.3 (CH_2_). ^19^F-NMR (CDCl_3_): δ -115.5 (s, 1F). **UPLC-MS**: Rt: 2.08 min, MS (ESI)^+^: 295.0 [M+H]^+^.

***N*-(4-Fluorobenzyl)-*N*-phenyl-1*H*-pyrrole-3-carboxamide (12b)**

Yield 89%, brown solid. ^1^H-NMR (CDCl_3_): δ 8.38 (bs, 1H, NH), 7.25-7.15 (m, 5H, H-aromatic), 6.99-6.95 (m, 2H, H-aromatic), 6.89-6.84 (m, H-aromatic), 6.49-6.46 (m, 1H, H-aromatic), 6.40-6.38 (m, 1H, H-aromatic), 5.74-5.71 (m, 1H, H-aromatic), 4.93 (s, 2H, CH_2_). ^13^C-NMR (CDCl_3_): δ 165.2 (C=O), 163.0 (d, J_C-F_= 245.0 Hz, C), 143.5 (C), 133.7 (d, J_C-F_= 3.2 Hz, C), 130.5 (d, J_C-F_=8.0 Hz, CH), 129.3 (CH), 128.9 (CH), 127.6 (CH), 123.8 (CH), 119.1 (C), 117.4 (CH), 115.2 (d, J_C-F_= 21.3 Hz, CH), 110.6 (CH), 53.3 (CH_2_). ^19^F-NMR (CDCl_3_): δ -115.5 (s, 1F). **UPLC-MS**: Rt: 1.77 min, MS (ESI)^+^: 325.1 [M+H]^+^.
