# Supplementary figures and images for: Rational design of highly potent broad-spectrum enterovirus inhibitors targeting the nonstructural protein 2C
Source: PLoS Biol. 2020 Nov 6;18(11):e3000904. doi: 10.1371/journal.pbio.3000904 (PMC7673538; doi:10.1371/journal.pbio.3000904)

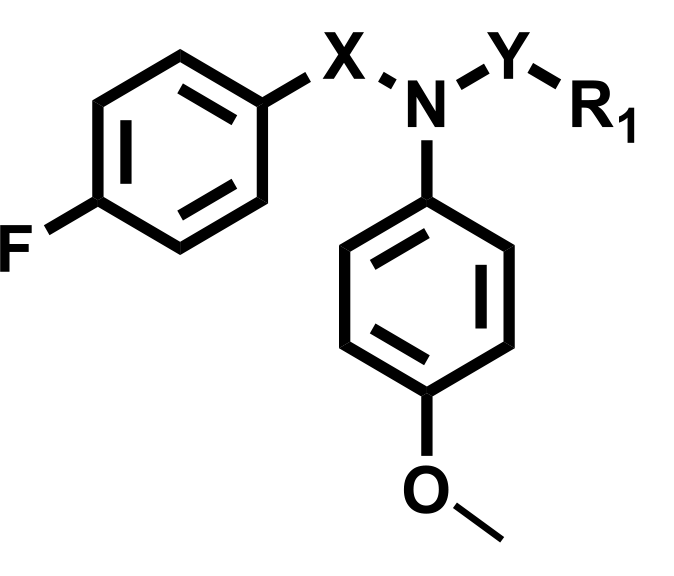


| **Compound** | **X** | **Y** | **R1** | **EC50(μM)** | **CC50(μM)** |
| --- | --- | --- | --- | --- | --- |
| **1** | CH2 | CO |  | 1.92 ± 0.04 | >30 |
| **5a** | CH2 | CO |  | >30 | >30 |
| **5b** | CH2 | SO2 |  | >30 | >30 |
| **5c** | CH2 | CO |  | 3.01 ± 0.2 | >30 |
| **5d** | CH2 | CO |  | 0.51 ± 0.04 | >30 |
| **5e** | CH2 | CO |  | 6.44 ± 0.42 | >30 |
| **5f** | CH2 | CO |  | >30 | >30 |
| **5g** | CH2 | CH2 |  | >30 | >30 |
| **6** | CH2 | CO |  | >30 | >30 |
| **12a** | CH2 | CO |  | 0.08 ± 0.02 | >30 |
| **15** | C=O | CH2 |  | >30 | >30 |

Supplement: S1 Table — Multicycle viral replication assays were performed in HeLa R19 cells, and shown are EC50 and CC50 values in μM. Data represent mean ± SD calculated from two different experiments both performed in biological triplicates. All underlying experimental data are displayed in S1 Data. (DOCX) [file pbio.3000904.s004.docx]

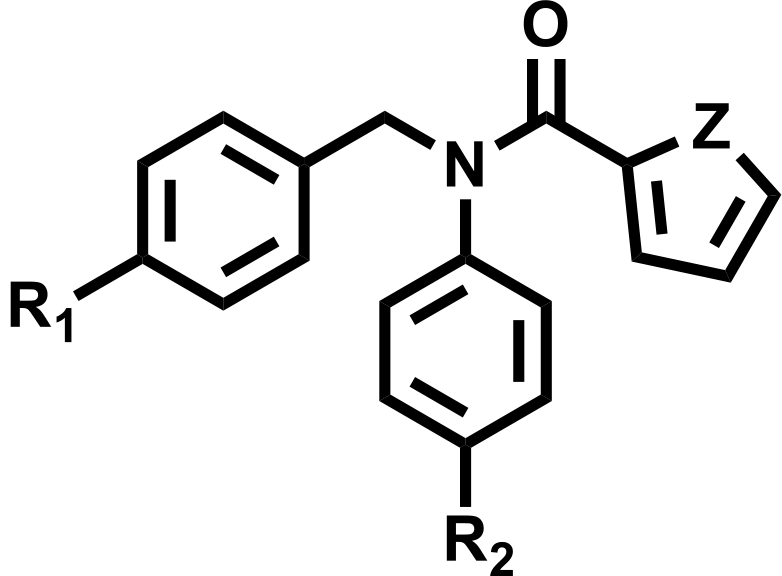


| **Compound** | **R1** | **R2** | **Z** | **EC50(μM)** | **CC50(μM)** |
| --- | --- | --- | --- | --- | --- |
| **1** |  |  | O | 1.92 ± 0,28 | >30 |
| **19a** |  |  | O | 0.91 ± 0.03 | >30 |
| **19b** |  |  | O | 0.83 ± 0.10 | >30 |
| **19c** |  |  | O | 4.07 ± 0.25 | >30 |
| **19d** |  |  | O | 0.79 ± 0.06 | >30 |
| **19e** |  |  | O | 0.86 ± 0.12 | >30 |
| **19f** |  |  | O | 3.10 ± 0.42 | >30 |
| **19g** |  |  | O | 0.88 ± 0.02 | >30 |
| **19h** |  |  | O | 7.03 ± 0.92 | >30 |
| **19i** |  |  | O | 2.45 ± 0.10 | >30 |
| **22** |  |  | NH | 1.50 ± 0.08 | >30 |

Supplement: S2 Table — Multicycle viral replication assays were performed in HeLa R19 cells, and shown are EC50 and CC50 values in μM. Data represents mean ± SD calculated from two different experiments both performed in biological triplicates. All underlying experimental data are displayed in S1 Data. (DOCX) [file pbio.3000904.s005.docx]

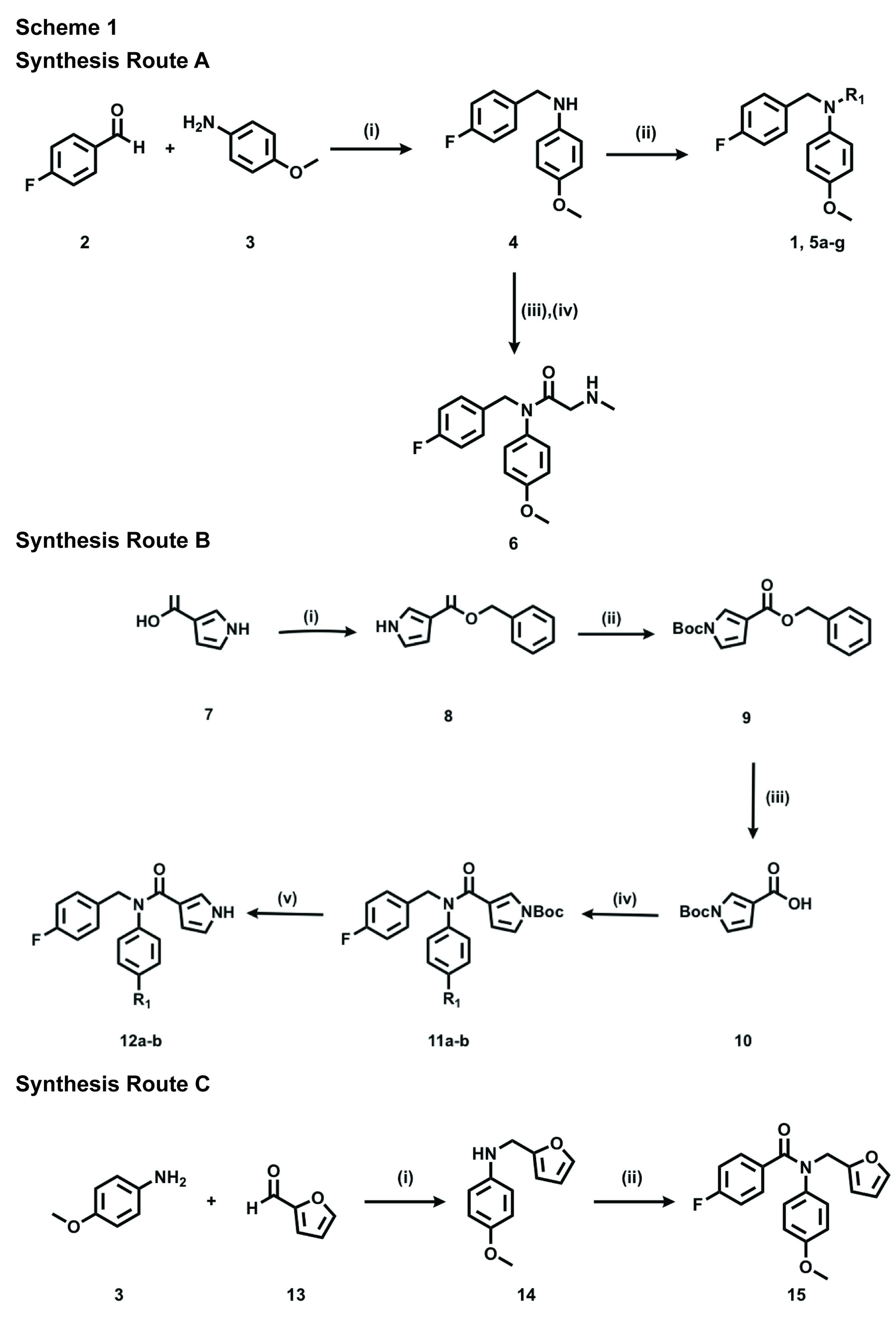

Supplement: S1 Fig — Synthetic route a): (i) NaBH4, MeOH/THF (4:1), rt, 6 h, quantitative; (ii) Compounds 5a–5d: corrisponding acyl chloride or sulphonyl chloride, TEA, DCM, rt, 3h, 63%–98%; Compounds 1, 5e–f: corrisponding carboxylic acids, TBTU, DIPEA, DMF, rt, on, 37%–69%; Compounds 5g: Na(AcO)3BH, MeOH, rt, on, 30%; (iii) 2-bromoacetyl chloride, TEA, DCM, rt, 1h, 68%; (iv) methylamine, EtOH, rt, on, 66%; Synthetic route b): (i) K2CO3, benzyl bromide, DMF, rt, 18h 68%; (ii) Boc, DMAP, TEA, THF, rt, 18h, 90%; (iii) Pd/C 10%, H2 atmosphere, EtOAc/MeOH (1:1, rt, 20h, 93%; (iv) compound 4 or compound 18b, TBTU, DIPEA, DMF, 45 °C, 48–72h, 79%–45%; (v) DCM/TFA (1:1), rt, 4h, 91%–89%. Synthetic route c): (i) NaBH4, MeOH/THF (4:1), rt, 6 h, 88%; (ii) 4-fluorobenzoyl chloride, TEA, DCM, rt, 3h, quantitative. (TIF) [file pbio.3000904.s008.tif]

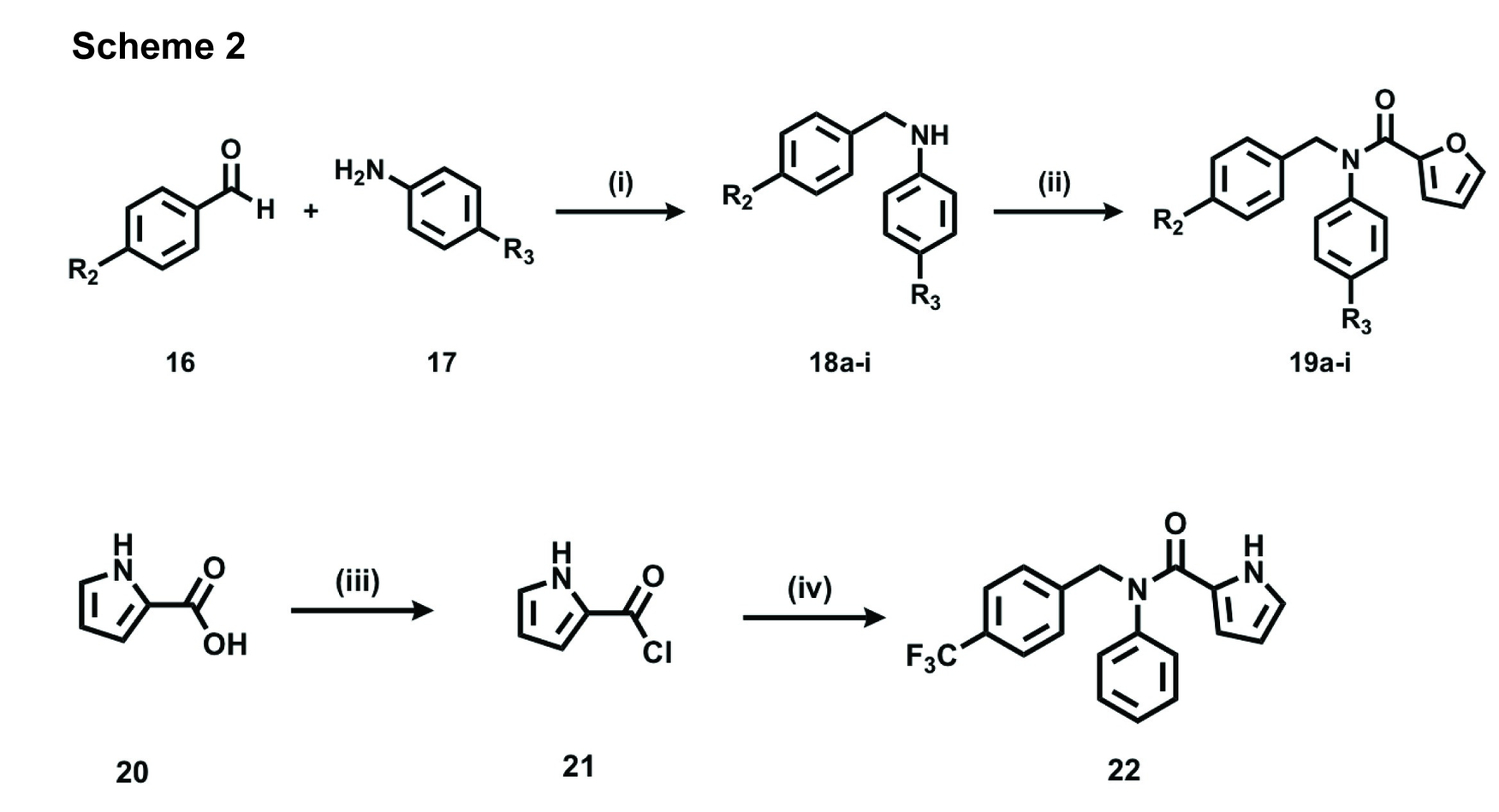

Supplement: S2 Fig — (i) NaBH4, MeOH/THF (4:1), rt, 6 h, 40%–99%; (ii) furan-2-carbonyl chloride, TEA, DCM, rt, 3 h, Y = 56%–99%; (iii) thionyl chloride, DCM, reflux, 2h; (iv) compound 18d, TEA, DCM, rt, 3h, 75%. (TIF) [file pbio.3000904.s009.tif]

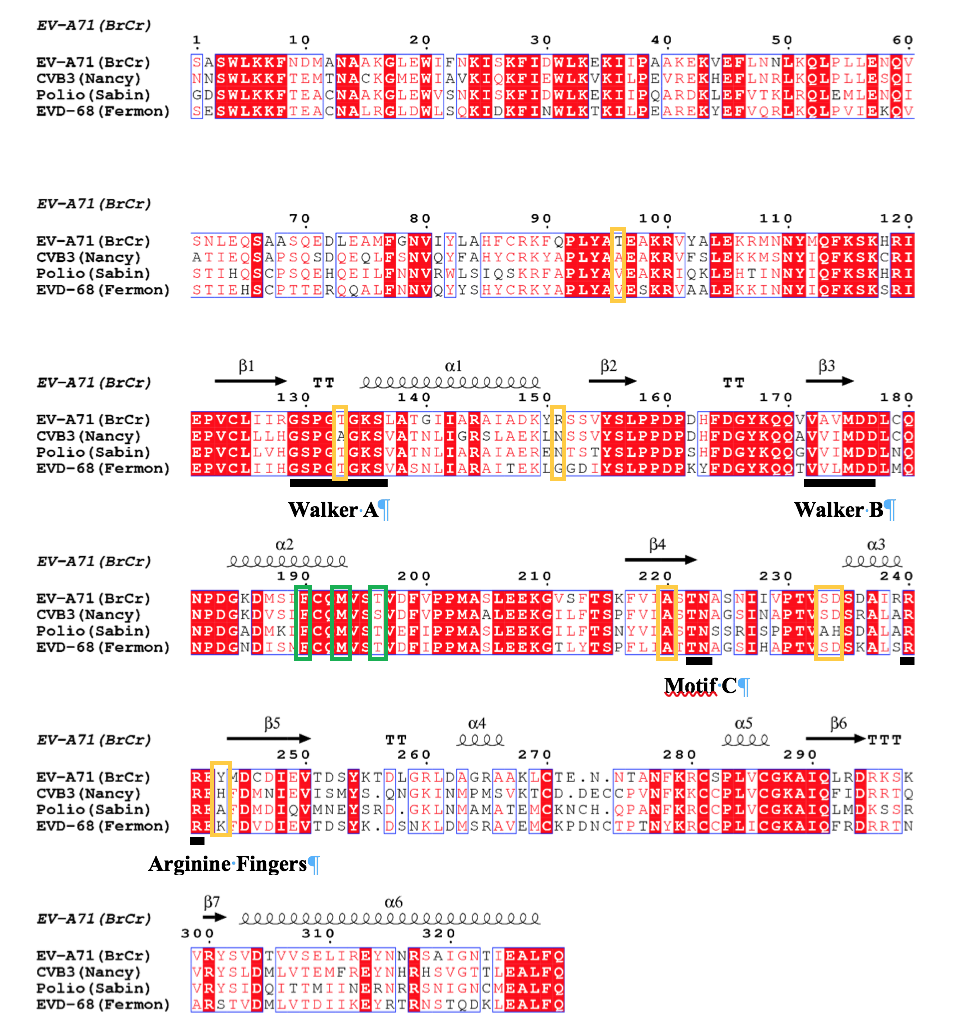

Supplement: S3 Fig — Multiple sequence alignment of EV-A71 (strain BrCr), CV-B3 (strain Nancy), PV (strain Sabin), and EV-D68 (strain Fermon) was performed with Clustal Omega. Invariant amino acids are highlighted in red. Secondary structural elements are shown on top of the alignment and are based on the EV-A71 crystal structure (PDB: 5GRB). Functional motifs are indicated in black. The green box indicates resistance mutations which are located at or close by the α2 helix. The yellow boxes highlight distal mutations. (TIF) [file pbio.3000904.s010.tif]

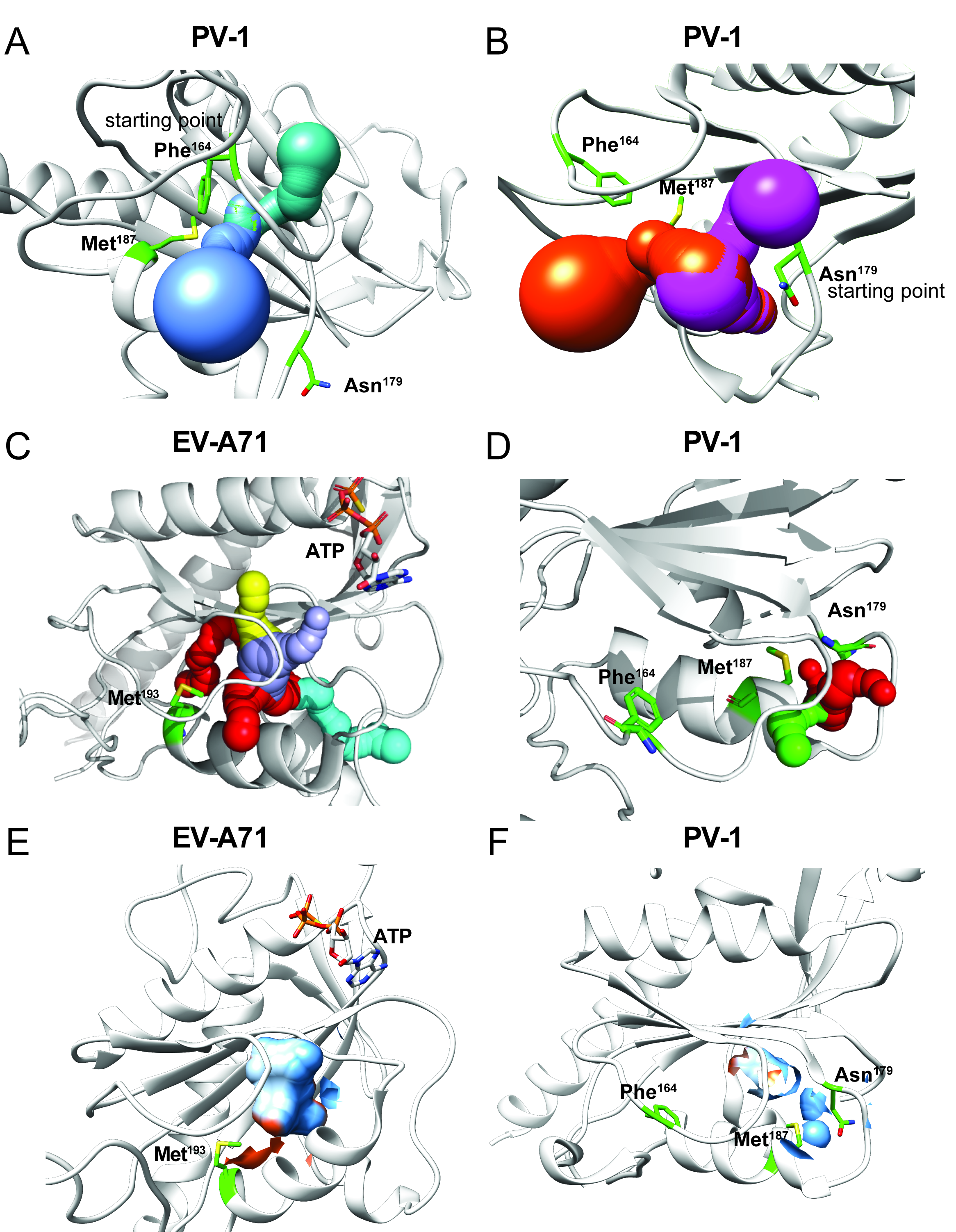

Supplement: S4 Fig — The mole online version (40) was used to calculate solvent exposed tunnels in 2C of PV-1 (PDB: 5z3Q) from different starting points. (A) The starting point for calculation was the amino acid F164, and in (B) the starting point for the tunnel prediction was N179. The Pymol plug-in CAVER 3.0.3 was used to calculate solvent accessible tunnel in the nonstructural protein 2C (58). (A) The EV-A71 2C crystal structure PDB: 5GRB, chain B was used. The amino acid M193 in the α2 helix of 2C is depicted in green and represents the starting point to identify solvent accessible tunnels. The identified tunnels are shown red, blue, green, and yellow. (B) For PV, the 2C crystal structure 5Z3Q, chain B was used to identify solvent accessible tunnels using the amino acid M187 as starting point. The identified tunnels are depicted in red, green, and blue. The CASTp online tool was used to predict cavities on the protein surfaces of the 2C nonstructural proteins of (C) EV-A71 and (D) PV (59). The cavities are highlighted in blue. (TIF) [file pbio.3000904.s011.tif]

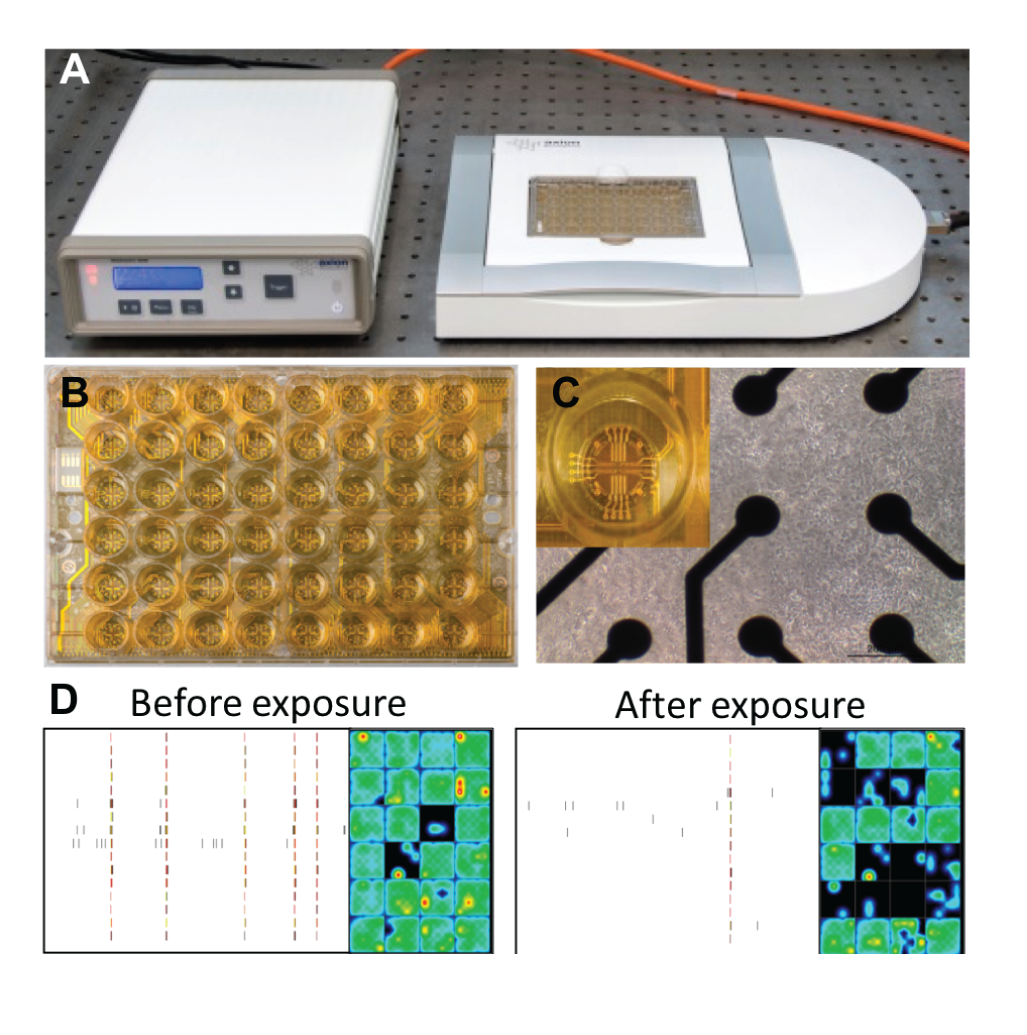

Supplement: S5 Fig — (A) Schematic overview of the microelectrode array (MEA) recordings used to measure changes in neuronal activity. Photographs of the Maestro 768-channel amplifier (A) and 48-well MEA plate (B). Each well contains 16 nanotextured gold micro-electrodes on top of which neuronal cells can be cultures for recording of spontaneous electrical activity (C). Baseline activity recorded before exposure (D, left) is compared to activity following exposure to a (inhibitory) test compound (D, right) to determine a TR that describes the changes in neuronal activity due to exposure to the test compounds. Modified after Tukker and colleagues, 2016 [55]. TR, treatment ratio. (TIF) [file pbio.3000904.s012.tif]

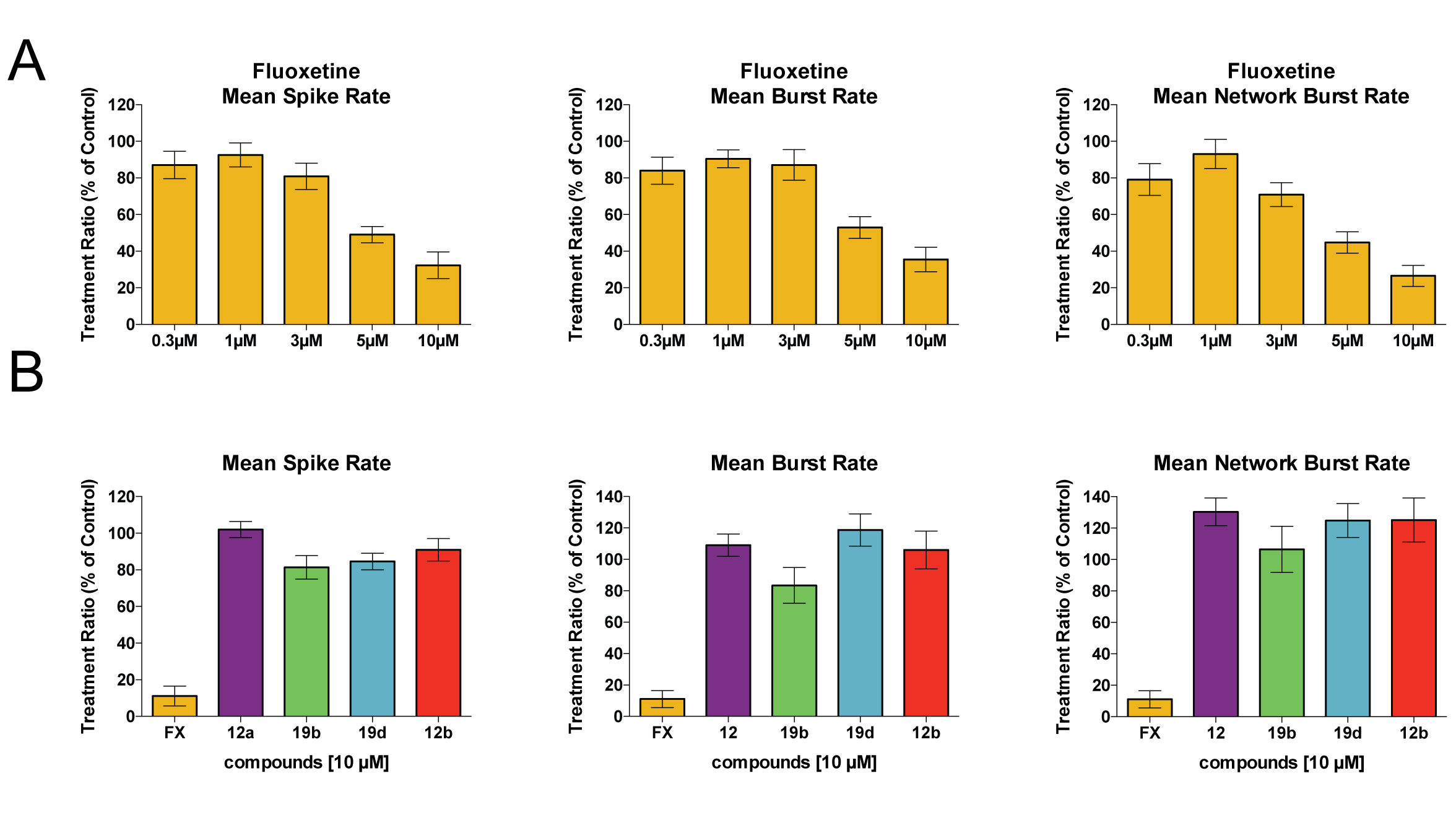

Supplement: S6 Fig — (A) Modulation of spontaneous neuronal activity by fluoxetine. Concentration–response curves for inhibition of MSR (left), MBR (middle), and MNBR (right) following acute exposure to fluoxetine. Neuronal activity is depicted as the mean TR ± SEM as percent of control (DMSO) wells (n = 12–16 wells, derived from 2 independent cultures, *P < 0.05). (B) Modulation of spontaneous neuronal activity by fluoxetine and antiviral compounds. When tested at a single, high concentration (10 μM), fluoxetine induced a profound inhibition of MSR (left), MBR (middle), and MNBR (right), whereas the antiviral compounds 12a, 12b, 19b, and 19d were without effect. Neuronal activity is depicted as the mean TR ± SEM as percent of control (DMSO) wells (n = 8–16 wells, derived from 2 independent cultures, *P < 0.05). All underlying experimental data that are displayed can be found in S2 Data. TR, treatment ratio. (TIF) [file pbio.3000904.s013.tif]
