# Supplementary material for: Rational design of highly potent broad-spectrum enterovirus inhibitors targeting the nonstructural protein 2C
Source: PLoS Biol. 2020 Nov 6;18(11):e3000904. doi: 10.1371/journal.pbio.3000904 (PMC7673538; doi:10.1371/journal.pbio.3000904)
Supplement: Attachment — Submitted filename: 20200707_ResponsetoReviewers.docx [file pbio.3000904.s014.docx]

**Manuscript ID:** PBIOLOGY-D-20-01439R1

**Title:** Rational design of highly potent pan-enterovirus inhibitors targeting the non-structural protein 2C

**Author(s):** Lisa Bauer, Roberto Manganaro, Birgit Zonsics, Daniel L. Hurdiss, Marleen Zwaagstra, Tim Donselaar, Naemi G.E. Welter, Regina G.D.M. van Kleef, Moira Lorenzo Lopez, Federica Bevilacqua, Thamidur Raman, Salvatore Ferla, Marcella Bassetto, Johan Neyts, Jeroen R.P.M. Strating, Remco H. S. Westerink, Andrea Brancale, Frank J.M. van Kuppeveld

**Journal:** PLOS Biology

Response to Reviewer #1

Picornaviruses are significant human pathogens for which there is no vaccine or antivirals. This manuscript described the lead optimization based on compound 1, which was identified through a HTS. Several analogs including 12, 19b and 19d, were identified to have broad-spectrum antiviral activity against EV-A71, EV-D68, CVA24, Polio and HRV. The mechanism of action was mainly studied by the resistance-selection experiment. Additional evidence was provided by the thermal shift binding assay. The highlights of this study include:

1)	Broad-spectrum antiviral profiling against multiple enteroviruses.

2)	Detailed pharmacological characterization. Using compound 19d as a chemical probe, resistant mutants have been selected in CV-B3, EV-A71 and EV-D68 viruses. Mutants were identified in the 2C protein, and the corresponding mutant viruses were generated through reverse genetics.

Although the leads generated from this study are promising drug candidates for picornavirus antiviral, there are several issues that remain to be addressed:

1)	The antiviral activity of the optimized hits 12,19b and 19d are on par similar to the (S)-fluoxetine (Table 5), although it is noted that the antiviral spectrum is expanded. The potency needs to be further optimized down to sub-micromolar to have clinical relevance. There are several series of 2C inhibitors in the literature that have submicromolar efficacy. Therefore, the results in this study are not particularly significant.

We combined the chemical features of the inhibitor 12a and 19d. We evaluated the antiviral activity of compound 12b in multicycle viral replication assays against the panel of EV. The compound 12b showed a further improvement in antiviral activity and inhibits some enteroviruses now in the nM range! We included these experiments in the new Figure 7.

2)	The detailed mechanism of action at the molecular level remains elusive. Although resistant mutants have been identified, no docking models were shown to illustrate how compound 19d interact with the 2C protein. The results shown in Fig. 6 have no direct relationship with the drug binding. Overall the detailed mechanism of action remains a mystery and this study does not significantly advance our knowledge on the pharmacology of 2C inhibitors.

This study identifies novel improved inhibitors and provides the first insight into their mode of action by showing that the predicted binding pocket is not directly in active site of 2C but rather on the opposite site from where the ATP is coordinated. Thus, this indicates that the 2C inhibitors target the 2C protein allosterically.

3)	The authors did not explain from the structural perspective why compounds 12, 19b and 19d have a broader spectrum of antiviral activity compared to fluoxetine.

At the present time, there is no structural information available that would give a reasonable indication on how these compounds will dock on the 2C protein. Although we have put forward an hypothesis on where the binding pocket might be, based on the resistance profile observed, a docking simulation will still lead to highly speculative results that would not necessarily add useful information to the manuscript. For this reason, we would prefer not to include such calculations in the paper.

4)     The overall approach is not particularly novel, it represents a standard medicinal optimization approach. Therefore, this manuscript is more suitable for chemistry journals.

The focus of the manuscript was the biological evaluation of the compounds and not the chemical synthesis. We used the compounds as a tool to identify key residues of the common druggable binding pocket in 2C. By generating compound resistant viruses, we reveal that the binding pocket is not directly in active site of 2C but rather on the opposite site from where the ATP is coordinated. Thus, this indicates that the 2C inhibitors target the 2C protein allosterically. Thus, our manuscript provides important information on the molecular mode of action of the described inhibitors.

Specific comments are:

1)	It was claimed that one of the advantages of current hits is that they are not neuroactive. I am not sure what this actually means. Does this have to do with the potential side effects of fluoxetine analogs? The compounds described here are still at the early stage of drug development, and there are many parameters need to be optimized including microsomal stability, solubility, permeability, CYP inhibition and etc. Therefore, neurotoxicity side effect is not a major concern at this stage.

It is true that we are still at the early stage of drug development but given the reported side effects of fluoxetine, with which our compounds share resemblance, we think it is important to note the lack of neurotoxicity for the class of compounds described in this manuscript.

2)	Line 113, "CPE-reduction assays" should read "CPE assay". There is only plaque reduction assay, not CPE reduction assay. This should be changed throughout the manuscript.

We corrected in the whole manuscript CPE-reduction assay to CPE-based multicycle viral replication assay.

3)	In table 2, the structures for R1 substitution are missing.

We apologize for the mistake and edited the table and added the missing substitutions.

4)	In Fig. 7, why the other two mutants H243R, F190L+H243R have not been tested?

We don’t understand this question, as the mutants H243R and F190L+H243R are described in Figure 7A.

Response to Reviewer #2:

The authors report the identification of antiviral compounds with pan-enterovirus and pan-rhinovirus activity starting from a previously reported compound 1.  The escape mutant analysis has permitted the authors to suggest that enteroviruses and rhinoviruses could share a druggable pocket in the 2C protein that needs further validation.

Major comments:

Overall this is a difficult paper to read and fully appreciate the key finding proposed by the authors that there is a common druggable pocket in entero- and rhinoviruses that can be targeted to try and obtain potent drugs.  An alignment of 2C protein from the various strains examined in this study in relation to previous mutations in CV-B3 virus and the strains compared in this study may be useful for non-EV readers to try and integrate the findings in the different viruses.  Data on SSRI activity of the new compounds could add value to attractiveness of this compound which presumably needs to be further optimized for potency.

We provided a structure-based alignment of the different 2C proteins in Supplement Figure 2 and highlighted the conserved residues, the resistance mutations and the catalytic center. We added more references to the main text for supplement figure 2. Further, the SSRI activity (Figure 3) and neuroactivity assays for the pan-EV and RV inhibitors (Supplement Figure 4) were already included in the main text. Additionally, we added the SSRI activity of the new compound 12b in the main text. (see Figure 8)

Minor comments:

Table 2 is incomplete.

We apologize for the mistake and missing parts have now been incorporated

Line 42 : …numbered enteroviruses and rhinoviruses?

We added better explanation in line 42 numbered enteroviruses (e.g. EV-A71, EV-D68) and rhinoviruses (e.g. HRV-A2, HRB-14)

Line 335: … should it be  "mutants were not tested"

Correction was made

Line 714: … should it be "end-point dilution assay"

Correction was made

Response to Reviewer #3:

Enteroviruses are ubiquitous pathogens. Despite decades of trying, no direct acting antiviral medications exist. This is an extremely well written paper that describes the exploration of anti-enteroviral activities and cytotoxicity of derivatives of a compound described originally by Zuo et al (ref 20). The authors, noting the structural similarities between this compound and fluoxetine (shown by Dr. van Kuppeveld et al and Zuo et al to inhibit replication) carefully modified key elements of this molecule and in the process identified congeners with broader antiviral activity, reduced impact on neuronal activity and lower cytotoxicity.  The paper closes somewhat speculatively by analyzing models of the target of action of these compounds, the viral 2C protein.

The paper is highly detailed and may have initially been composed with the goal of publishing it in a medicinal chemistry journal. The detailed description of the schema for synthesis does not detract from the paper, and instead makes it all the more impressive. The only criticisms of this detail may be the complexity of the figure legends, and several small errors in labeling.

For example, in line 163 the text refers to "final product 9", which appears to refer to compound 15 (the final product of Synthesis route C).

We apologize for the mistake and made the correction.

Also, in two places "13d" (e.g. line  347) is mentioned, when 19d is intended.

We apologize for the mistake and made the correction.

Several other minor errors are present in Table legends for Table 4 and 5 with begin wiht "Multicycle (sic) was performed in Hela R19 cells,.."  (Multicycle replication assay were performed..." may have been intended).

Corrections were made.

Also, a number of terms and abbreviation may need definition for the audience of this journal.

We checked the text and added clarity where needed.

Two somewhat important points that should be addressed follow:

First, the authors should  define levels of antiviral activity. Most experts would not consider EC50 values of 5 to 10 micromolar to be more than mild to moderate; submicromolar is more typical of molecules that come to be used therapeutically. The paper would be improved by mentioning this or similar stratification.

We agree with the reviewer that mentioning the therapeutically relevant range would improve the paper. We defined the therapeutically relevant window in line 793-796.

One related point:  the EC-50 values for  compound 12 CV-B3 (Nancy) of ~10 to 20 nM are very impressive, but since this strain no longer circulates, one wonders why other enterovirus B virus strains were not examined, as was done with EV-A71, EV-D68, and CVA24.

We focused on clinical isolates from the serotypes EV-A71, EV-D68 and CV-A24v because in the last years large outbreak with these viruses have had a major clinical and socio-economic impact. Unfortunately, we had no access to clinical isolates of enterovirus B strains.

Addressing these two points would not detract from this excellent paper, but as it is, the authors seem a bit overly enthusiastic in their characterization of their best compounds as candidates for clinical development._____________________________________________
