# Supplementary material for: Rational design of highly potent broad-spectrum enterovirus inhibitors targeting the nonstructural protein 2C
Source: PLoS Biol. 2020 Nov 6;18(11):e3000904. doi: 10.1371/journal.pbio.3000904 (PMC7673538; doi:10.1371/journal.pbio.3000904)
Supplement: Attachment — Submitted filename: 20200828_response_to_reviewer.docx [file pbio.3000904.s015.docx]

Response to the reviewers:

Reviewer #3

Reviewer #3: The authors of this manuscript now submit a revised version that corrects the typographic errors in the first version, clarifies a number of points, and  most importantly, demonstrates the antiviral properties of a new  compound (12 b) resulting from the logical incorporation of properties of two derivatives of a molecule identified by another group (ref 20) via high-throughput screening. This new compound exhibits appreciable activity against representative strains from enterovirus species A, B, C, and D, and two of the 3 rhinovirus species. The paper is much improved by these changes.  However, several straightforward changes should be made, in this reviewers opinion.

1. The authors state in their responses that "we agree that mentioning the therapeutically relevant range (for EC50 values) would improve the paper. We defined the therapeutically relevant window in line 793-796". However, lines 793 to 796 are in the section labeled "MEA recordings of spontaneous neuronal activity in rat primary cortical cultures".  What is needed is a straight forward statement in the methods, such as saying that antiviral activity is defined as 50% inhibition of virus production or expression of luciferase at concentrations of 10 micromol/ L or less. Clearly submicromolar activity is typical of most antivirals in use,  and it is exciting to see examples of low nanomolar range reported for compound 12b..

We added several sentences into the Multicycle virus infection methods part. “*The concentration of compound that inhibits virus-induced cell death or expression of Renilla luciferase by 50% (50% effective concentration [EC_50_]) was calculated by nonlinear regression analysis with GraphPad Prims Version 6. We set the threshold for compounds activity at concentration of 10µM or less since low µM range is typical for antivirals in clinical use. Cytotoxicity of the compounds was assessed in a similar set-up, and 50% cytotoxic concentration (CC_50_) values were derived from cell viability values determined with an MTS assay*.” The changes were made in line 817-823. Line numbering corresponds to new uploaded track-changed manuscript.

2. In the introduction, the authors state "Even though fluoxetine was well tolerated, it revealed no clinical benefit. Rather, the data pointed to a worsening of the patient conditions in the fluoxetine-treated cohort.(26)  This is paradoxical; if people became more ill with fluoxetine, how can we say that it was well tolerated? Worsening of disease is obviously an adverse event to an antimicrobial agent.

We changed the sentence in the introduction to “The treatment with fluoxetine revealed no clinical benefit but rather suggested a worsening of the patient conditions in the fluoxetine-treated cohort .(26)” in line 83-85. Line numbering corresponds to new uploaded track-changed manuscript.

3. Line 635 states that mechanistically, "the energy produced by ATP hydrolysis induces a series of conformational changes which drive unwinding of RNA or DNA."  A reference should be provided that best supports this precise mechanistic statement, as until recently the helicase activity was in question, as it had been inconsistently demonstrated.

This comment was ment as generic statement for helicases from the SF3 superfamily in which the energy provided by the ATP hydrolysis causes conformational changes within the helicase which in turn allows the unwinding of a nucleic acid substrate. For clarification we modified the sentence “*The energy produced by ATP hydrolysis induces a series of conformational changes which drive unwinding of RNA or DNA by SF3 helicases.”* and additionally, we added the reference Singleton et al., 2007.The change corresponds to line 658 in the new uploaded track-changed manuscript.

4. Isn't it an overstatement to say declare in the Discussion that " several comments exhibited " pan-EV and -RV activity" line 581?  Pan clearly means "all" but the authors demonstrate data from only 2 of ~ 160 rhinovirus types, one-EV-B strain, and one EV-C strain (Sabin 1). Granted, at least activity is  being seen against strains from 6 of 7 species...but it is an overstatement to call this pan-EV activity, and a more modest claim is appropriate.

We agree with the reviewer that we overinterpreted our results and we changed the pan-EV activity into broad-spectrum activity throughout the manuscript.
